# Contrasting evolutionary origins of two mountain endemics: *Saxifraga wahlenbergii* (Western Carpathians) and *S. styriaca* (Eastern Alps)

**DOI:** 10.1186/s12862-019-1355-x

**Published:** 2019-01-11

**Authors:** Natalia Tkach, Martin Röser, Tomasz Suchan, Elżbieta Cieślak, Peter Schönswetter, Michał Ronikier

**Affiliations:** 1 Institute of Biology, Martin Luther University Halle-Wittenberg, Neuwerk 21, 06108 Halle, Germany; 2 W. Szafer Institute of Botany, Polish Academy of Sciences, Lubicz 46, 31-512, Krakow, Poland; 3 Department of Botany, University of Innsbruck, Sternwartestraße 15, 6020 Innsbruck, Austria

**Keywords:** Alps, Carpathians, Endemics, European mountains, Hybrid speciation, ITS genomic screening, Molecular clock, NGS, Phylogenetics, Tertiary relict

## Abstract

**Background:**

The Carpathians and the Alps are the largest mountain ranges of the European Alpine System and important centres of endemism. Among the distinctive endemic species of this area is *Saxifraga wahlenbergii*, a Western Carpathians member of the speciose genus *Saxifraga*. It was frequently considered a taxonomically isolated Tertiary palaeopolyploid and palaeoendemic, for which the closest relatives could not yet be traced. A recently described narrow endemic of the Eastern Alps, *S. styriaca*, was hypothesized to be closely related to *S. wahlenbergii* based on shared presence of peculiar glandular hairs. To elucidate the origin and phylogenetic relationships of both species we studied nuclear and plastid DNA markers based on multiple accessions and analysed the data in a wide taxonomic context. We applied Sanger sequencing, followed by targeted next-generation sequencing (NGS) for a refined analysis of nrITS variants to detect signatures of ancient hybridization. The ITS data were used to estimate divergence times of different lineages using a relaxed molecular clock.

**Results:**

We demonstrate divergent evolutionary histories for the two mountain endemics. For *S. wahlenbergii* we revealed a complicated hybrid origin. Its maternal parent belongs to a Western Eurasian lineage of high mountain taxa grouped in subsect. *Androsaceae* and is most likely the widespread *S. androsacea*. The putative second parent was most likely *S. adscendens*, which belongs to the distantly related subsect. *Tridactylites*. While Sanger sequencing of nrITS only showed *S. adscendens*-related variants in *S. wahlenbergii*, our NGS screening revealed presence of sequences from both lineages with clear predominance of the paternal over the maternal lineage.

**Conclusions:**

*Saxifraga styriaca* was unambiguously assigned to subsect. *Androsaceae* and is not the sister taxon of *S. wahlenbergii*. Accordingly, the similarity of the glandular hairs observed in both taxa rests on parallelism and both species do not constitute an example of a close evolutionary link between the floras of the Western Carpathians and Eastern Alps. With the origin of its paternal, *S. adscendens*-like ITS DNA estimated to ca. 4.7 Ma, *S. wahlenbergii* is not a relict of the mid-Tertiary climate optimum. Its hybrid origin is much younger and most likely took place in the Pleistocene.

**Electronic supplementary material:**

The online version of this article (10.1186/s12862-019-1355-x) contains supplementary material, which is available to authorized users.

## Background

The Carpathians constitute an important part of the European Alpine System (EAS sensu Ozenda [1]), which includes the Alps, the Pyrenees, the Apennines, and the mountain ranges of the northern Balkan Peninsula. Their location in the middle of the European continent causes richness of biota due to overlapping alpine, arctic-alpine, Mediterranean and Asian elements. Because only the highest massifs of the Carpathians were extensively glaciated during the glacial periods of the Pleistocene (see [2–4]), this range is considered an important northern glacial refugium [3, 5, 6]. Along with the Alps, the Carpathians form the largest and biogeographically important part of the EAS. Topography and climate of mountain systems are known to generally favour evolution of endemism [7] and, accordingly, these two ranges belong to important centres of endemism in Europe [8]. The number of endemics in the Carpathians is estimated at 3–5% of the vascular flora of the area (excluding apomictic complexes [3, 9]). In the Alps, it is higher and reaches 12.6% [10, 11].

**Fig. 1:**
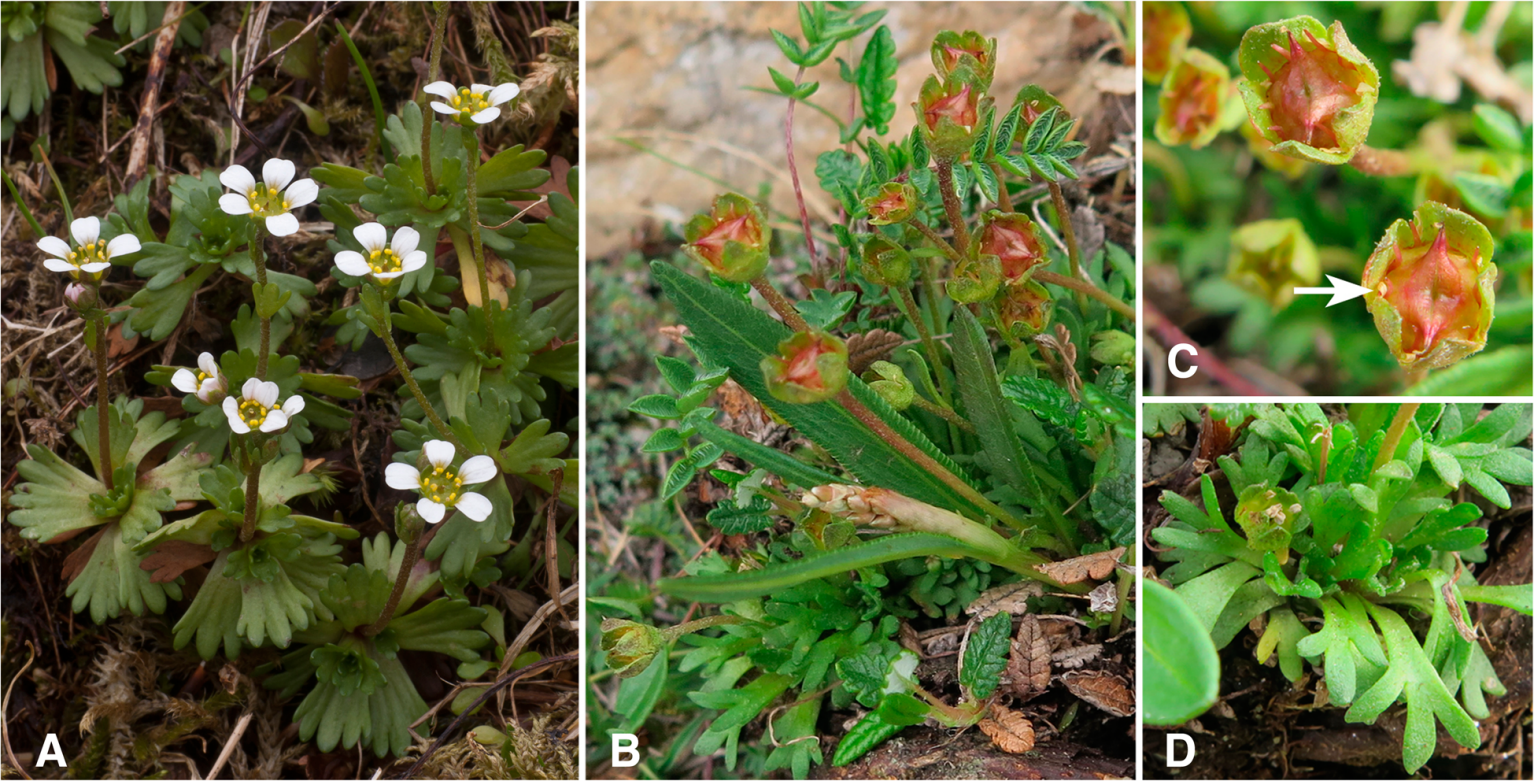
Photographs of *Saxifraga wahlenbergii* (**a**) and *S. styriaca* (**b**–**d**). **a** and **b** Habitus. **c** Flowers after anthesis. One of the tiny petals is marked by an arrow. *D* Rosette leaves. Photographs by S. Wróbel (**a**; Poland, Western Carpathians, Tatry, Wyżnia Świstówka, 14 June 2008) and N. Tkach (**b**–**d***;* Austria, Lower Tauern, Rettlkirchspitze, M. Röser 11,356 & N. Tkach, 16 June 2018)

**Fig. 2:**
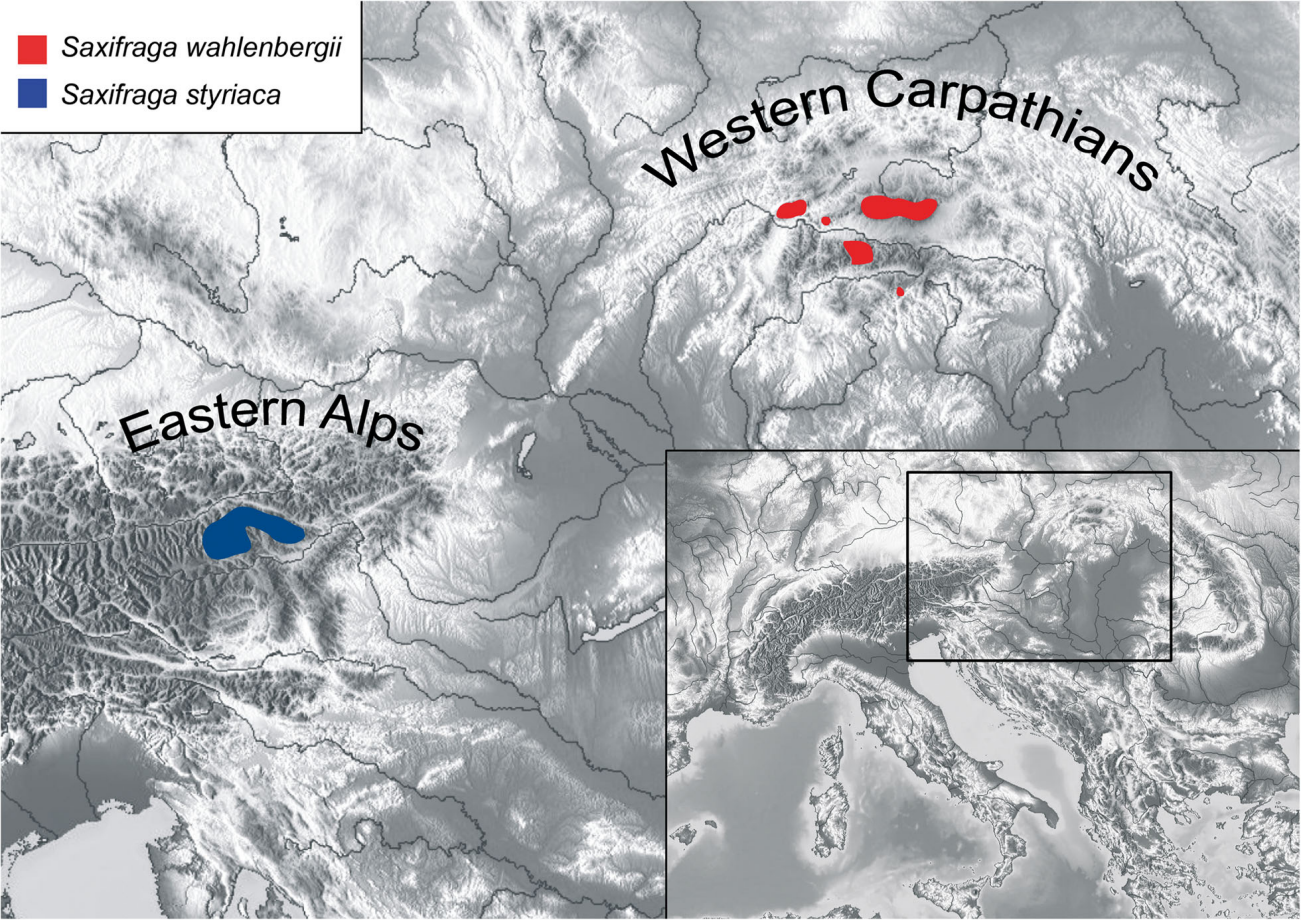
Distribution of *Saxifraga wahlenbergii* in the Western Carpathians and *S. styriaca* in the Eastern Alps modified from Köckinger (2003) and Jasičová & Futák (1985). Topographic map from OpenStreetMap contributors, https://maps-for-free.com/

In the European mountains, the genus *Saxifraga* is one of the most endemic-rich plant genera and, notably, it contributes most endemic taxa in the alpine and nival belts [12]. Many species of *Saxifraga*, representing various infrageneric units, occur in restricted areas throughout the EAS [13]. Although in the Carpathians the general taxonomic richness of this genus is much lower compared to the Alps (e.g., [14]), *S. wahlenbergii* Ball is counted among the most distinctive endemic plants of this mountain system (Fig. 1). It occurs only in the Western Carpathians, a significant centre of plant endemism (Fig. 2) [4, 8]. Its distribution is limited to the Tatra Mountains and a few neighbouring massifs [15]. Despite the attention this species has attracted, its taxonomic and biogeographic relationships remained rather obscure. Engler & Irmscher [16], followed by Pawłowski [17], considered *S. wahlenbergii* a distinct relict of the Tertiary flora, mostly on account of its presumed close relationship to the Pyrenean endemic *S. praetermissa*. Later, Pawłowska [18] contradicted the apparent morphological similarity evoked to place the two species in the same ‘grex’ or series. However, she maintained the position of the Carpathian taxon as a Tertiary palaeoendemic based on its assumed taxonomic isolation together with its restricted geographical range. In a cytological analysis of the flora of the Tatra Mountains, Skalińska [19] considered *S. wahlenbergii* a palaeopolyploid. Although she did not explicitly discuss the species’ age, it was suggested to belong to ‘very ancient groups’, which early accomplished their cytological differentiation.

**Table 1:** Summary of the stem and crown ages and statistics of the clades of *Saxifraga*

Node	Clade	Mean	Stderr of mean	Stdev	Median	Geometric mean	95% HDP interval	Support (ML/MP/PP)
1	Ingroup crown	92.6421	0.0773	4.4733	92.3832	93.6433	[89.1248, 102.3174]	100/100/100
2	Saxifragaceae stem	87.5454	0.1422	6.2863	87.2865	86.9088	[73.7372, 99.5412]	98/100/100
3	Saxifragaceae crown/*Saxifraga* stem	74.7372	0.3107	6.3138	74.4783	74.1812	[61.1301, 86.5916]	100/99/100
4	*Saxifraga* crown	56.4942	0.3444	6.3656	56.2354	55.8262	[43.5702, 68.0993]	100/98/100
5	sect. *Saxifraga* stem	28.6432	0.2688	4.0574	28.3844	28.2295	[21.2255, 37.1037]	100/96/100
6	sect. *Saxifraga* crown	22.7994	0.2181	3.4982	22.5405	22.4905	[16.3297, 29.9462]	100/100/100
7	*S. wahlenbergii*/*S. adscendens*/*S. tridactylites* crown	15.6867	0.1515	3.6955	15.4279	15.1955	[8.6162, 23.0655]	82/55/100
8	*S. tridactylites* crown	2.4579	0.038	1.3763	2.1818	2.1728	[0.443, 5.2431]	100/100/100
9	*S. wahlenbergii*/*S. adscendens* crown	5.5559	0.0815	1.8643	5.2632	5.3004	[2.5486, 9.3564]	100/97/100
10	*S. wahlenbergii* stem	5.284	0.0763	1.7883	4.7755	5.0103	[2.3751, 8.9502]	−/−/−
11	*S. wahlenbergii* crown	3.184	0.0567	1.7872	2.9251	3.3449	[1.2114, 5.4784]	72/61/97
12	subsect. *Androsaceae* stem	12.1632	0.1342	2.6315	11.8828	11.8872	[7.5249, 17.5502]	85/−/100
13	subsect. *Androsaceae* crown	9.3539	0.1134	2.2638	9.095	9.186	[5.5857, 13.7611]	72/62/96
14	*S. androsacea* & Co. crown	4.1407	0.0585	1.56	3.8818	3.8615	[1.6046, 7.2978]	98/86/100
15	*S. styriaca*/*S. seguieri*/*S. italica*/*S. depressa* crown	6.9361	0.0865	1.8087	6.6773	6.7081	[3.7057, 10.3506]	52/72/100
16	*S. seguieri*/*S. italica*/*S. depressa*/*S. androsacea* crown	5.8061	0.0667	1.7944	5.5473	5.9792	[2.9864, 9.0932]	−/−/57
17	*S. italica*/*S. depressa*/*S. androsacea* crown	3.968	0.01512	1.9095	3.7092	4.0365	[1.2059, 6.9024]	59/61/91
18	*S. italica*/*S. androsacea* crown	1.0901	0.0128	0.8246	0.89	0.804	[0.0213, 2.7148]	95/85/100
19	*S. seguieri* crown	1.8507	0.0217	1.1977	1.5919	1.5042	[0.1681, 4.2308]	88/68/100
20	*S. styriaca* crown	3.0915	0.037	0.12199	2.8326	2.7906	[1.0556, 5.4707]	96/97/100

The clades 1–20 are marked in Fig. 5 and Fig. 6

*Mean* Mean age, *Stderr of mean* standard error of mean age, *Stdev* Standard deviation, *Median* Median age, *Geometric mean* Geometric mean of age, *HDP* Highest posterior density, *ML/MP/PP* Maximum likelihood/Maximum parsimony/Posterior probability

*Saxifraga wahlenbergii* is characterised by peculiar pluricellular glandular hairs with wormlike endings, which induced Pawłowska [18] to accommodate it in the monotypic series *Perdurantes* (S.Pawł.) S.Pawł. Recently, this specific type of glandular hairs has also been observed in the newly described *S. styriaca* Köckinger, a narrow endemic of the eastern-most Eastern Alps (Lower Tauern), a region harbouring several rare and disjunctly distributed species (Figs. 1 and 2) [20, 21]. This species was therefore suggested to be a putative closest relative of the Carpathian endemic [22]. Both orophytes are characteristic of high altitudinal zones and have their upper distribution limit in alpine and subnival regions (2400–2500 m a.s.l.). *Saxifraga wahlenbergii* covers a much wider altitudinal range than *S. styriaca*, with the lower distribution limits around 800 m and 1860 m, respectively [22, 23]. While *S. styriaca* prefers base-rich schists, *S. wahlenbergii* is found on both calcareous and siliceous rocks [13, 22, 24]. A phylogenetic analysis testing whether the two species represent a phylogenetically separate lineage within the genus would be vital to unravel their evolutionary history. It would also contribute to the knowledge of evolution of endemic high mountain taxa and of the biogeography of the EAS.

If the close phylogenetic relationships of *S. wahlenbergii* and *S. styriaca* were confirmed, this species pair would provide an important case of floristic and evolutionary links between the Carpathians and the Alps. The floras of both mountains have close connections as reflected, for example, by the presence of common endemic species such as *Hypochaeris uniflora* Vill. and *Pinus cembra* L. [25–27] and vicariant species from many genera, such as *Cochlearia* L.*, Sempervivum* L. and *Soldanella* L. [28–31]. Biogeographical links are particularly strong between the Carpathians and the Eastern Alps including a large group of common species absent from the Western Alps, for instance *Campanula alpina* Jacq., *Dianthus glacialis* DC., *Doronicum stiriacum* (Vill.) Dalla Torre, *Gentiana frigida* Haenke and *Saponaria pumila* Janch. [17, 32–37]. Plant populations of these regions often display close phylogeographical connections as demonstrated, for example, in the *Arabidopsis arenosa* (L.) Lawalrée species complex, *Pritzelago alpina* (L.) O.Kuntze, *Ranunculus alpestris* L., *R. glacialis* L., *Rhodiola rosea* L. or *Salix herbacea* L. [38–43].

Despite their pertinence for the knowledge on biogeography and evolution of the Central European mountain flora, the relationship and phylogenetic position of *S. wahlenbergii* and *S. styriaca* have not been comprehensively studied so far. In a preliminary report, Cieślak et al. [44] placed both species in sect. *Saxifraga* and suggested their phylogenetic divergence. In a genus-wide molecular-phylogenetic analysis of *Saxifraga*, Tkach et al. [45] covered only a single accession of *S. wahlenbergii* and supported its sectional placement. Furthermore, due to discordance between the topologies of nuclear and plastid DNA trees, a hybrid origin of this taxon was hypothesised.

In this study, we aim at exploring the relationship of *S. wahlenbergii* and *S. styriaca* and their parentage in more depth. We apply molecular methods including Sanger sequencing and targeted next-generation sequencing (NGS) for a refined analysis of nuclear and plastid DNA variation. We use a range-wide sample of accessions of *S. wahlenbergii* and narrowly distributed *S. styriaca*, a broad phylogenetic context and temporal calibration of the phylogeny, to address the following questions: (1) Are *S. wahlenbergii* and *S. styriaca* closely related and eventually sister taxa, corroborating the morphology-based hypothesis? (2) What are their closest extant relatives? (3) What are the similarities and differences in the evolutionary history of both taxa? (4) Do they support a close biogeographical link between the floras of the Western Carpathians and the Eastern Alps? (5) Is the traditional hypothesis that *S. wahlenbergii* is a Tertiary relict phylogenetically supported?

## Methods

### Material

#### Sampling strategy

Our full dataset comprises 326 taxa. The 14 samples from 13 populations of *S. wahlenbergii* and four samples from two populations of *S. styriaca* were collected during field campaigns to the Carpathians and the Alps, respectively. To determine the phylogenetic position of *S. wahlenbergii* and *S. styriaca* within the genus *Saxifraga*, we added the newly generated sequences of these samples to the extensive plastid (*trn*L–*trn*F region) and nuclear ribosomal DNA (nrDNA; internal transcribed spacer region, ITS) data matrices generated for *Saxifraga* by Tkach et al. [45]. Additionally, further accessions of *S. adscendens*, *S. androsacea*, *S. aphylla*, *S. depressa*, *S. facchinii*, *S. hypnoides*, *S. praetermissa*, *S. seguieri* and *S. tridactylites* were studied. To increase phylogenetic tree resolution, an additional plastid non-coding intergenic spacer, *rpl*32–*trn*L, which was found informative in an earlier pilot study (E. Cieślak, M. Ronikier, unpubl. data), was studied for all taxa. These large (hereafter: ‘extended’) plastid and nr DNA datasets were rooted with *Itea virginica* L. and *Pterostemon rotundifolius* Ramírez, both belonging to Iteaceae J.Agardh.

For a better overview of phylogenetic relationships in the target group, reduced plastid and nuclear datasets including 56 taxa were used. They encompassed *S. wahlenbergii*, *S. styriaca,* their closest relatives (*S.* sect. *Saxifraga*; see Results) and only a few representatives of other *Saxifraga* sections as identified in the analyses of the extended datasets. *Micranthes nivalis* (L.) Small was chosen as outgroup.

A further dataset was created to enable an in-depth analysis of variants, intra-individual variation and interspecific sharing patterns based on the ITS2 region of nrDNA. Here, we employed next-generation sequencing (NGS) of amplicons to efficiently retrieve all distinct ITS2 copies present in the genomes. In the analysis, NGS data were combined with the reduced nrDNA dataset based on Sanger sequences (see above).

For the divergence time estimation, sect. *Saxifraga* was analysed within the broad phylogenetic framework of Saxifragales using this ITS dataset. It was extended to incorporate other representatives of the families Saxifragaceae, Iteaceae and Grossulariaceae and rooted with *Penthorum chinense* Pursh (Penthoraceae Rydb. ex Britton), a remotely related taxon from the order Saxifragales Bercht. & J. Presl [46, 47].

The species and accessions studied, voucher information and GenBank/ENA accession numbers for all used sequences (www.ncbi.nlm.nih.gov/genbank/; www.ebi.ac.uk/ena/) are listed in Additional file 1: Table S1. All sequence alignments are available in Additional file 2.

### Methods

#### Scanning electron microscope (SEM) analysis

Dried leaves were mounted under an incident light microscope (Zeiss, Germany) on aluminium stubs using double stick carbon conductive tabs (Plano GmbH, Wetzlar, Germany). Images of the uncoated samples were taken with the tabletop scanning electron microscope Hitachi TM-3030Plus (Hitachi Europe Ltd., Maidenhead, UK). The abaxial surface of the leaves and single trichomes were imaged using 15 kV in mixed signal mode (combined secondary and backscattered electron detectors). The following specimens were studied: *Saxifraga wahlenbergii* (Poland, Western Carpathians, Tatry Zachodnie Mt., R. Letz & P. Mráz, 7 Aug 2004, KRAM636368); *S. styriaca* (Austria, Alps, Lower Tauern, Rettlkirchspitze, M. Röser 11,356 & N. Tkach, 16 June 2018, HAL).

#### DNA extraction, PCR amplification and Sanger sequencing

Total genomic DNA was extracted from silica-gel dried plant material and herbarium specimens (Additional file 1: Table S1) using either the NucleoSpin Plant II DNA extraction kit (Macherey-Nagel, Düren, Germany) or the DNeasy Plant Mini Kit (Qiagen, Hilden, Germany), according to the manufacturers’ protocols.

Amplification and DNA sequencing using the Sanger chain-termination method of ITS was performed using the primers ITS-1, ITS-4 and ITS-5 of White et al. [48]. For amplification and sequencing of *trn*L–*trn*F including the *trn*L(UAA) intron and the adjacent intergenic spacer between the *trn*L(UAA) 3’exon and the *trn*F(GAA) gene, primers c, d, e and f from Taberlet et al. [49] were used. The *rpl*32–*trn*L region was amplified and sequenced using the primers *trn*L^(UAG)^ and *rpl*32-F [50].

For amplification of ITS and *trn*L–*trn*F we followed Tkach et al. [45] or applied a slightly different ITS protocol based on a touchdown cycling profile as follows: initial denaturation at 94 °C for 3 min, followed by 35 cycles of 30 s at 94 °C, 30 s at 60 °C (with decrease of 1 °C by cycle and constant temperature of 50 °C from the 11th cycle onwards), and 1 min at 72 °C, followed by a final extension at 72 °C for 7 min. The *rpl*32–*trn*L region was amplified using the following PCR program: initial denaturation at 94 °C for 2 min, followed by 34 cycles of 94 °C for 30 s, 53 °C for 1 min, and 72 °C for 2 min. The final extension at 72 °C lasted for 10 min.

The quality and quantity of the PCR products were checked spectrophotometrically or on 1% agarose gel. Sequencing of new *S. wahlenbergii, S. styriaca* and *S. adscendens* samples was carried out using BigDye 3.1 chemistry (Thermo Fisher Scientific, Waltham, Massachusetts, U.S.A.) and sequences were separated on an ABI 3130 automated sequencer. Sequencing of other samples was performed by LGC Genomics (Berlin, Germany).

The sequences were edited in Sequencher 5.0 (Gene Codes Corporation, Ann Arbor, Michigan, U.S.A.). The new ITS and *trn*L–*trn*F sequences were added to the alignments of Tkach et al. [45]. The sequences were automatically aligned using the default settings of ClustalW2 [51] implemented in Geneious 9.1.6 (www.geneious.com) [52]. Subsequently, the alignments were refined manually.

#### NGS library construction

Our NGS protocol largely followed Suchan et al. [53] and consisted of two PCR steps. First, the ITS2 region was amplified using a combination of ITS-S2F [54] and ITS-4R [48] primers, previously successfully used in barcoding [55] and metabarcoding studies [56, 57], tailed with a partial Illumina adapter. The second PCR reaction used double-indexing primers to uniquely tag sequences belonging to each specimen analysed. The first reaction consisted of 1 μl of the sample, 1× Q5 buffer, 0.4 U of Q5 Hot-Start polymerase (New England Biolabs, Ipswich, Massachusetts, U.S.A.), 200 μM of each dNTP, and 0.5 μM of each primer in a 10 μl reaction volume, and was amplified using 15 PCR cycles. Each sample was processed in two replicates. Each replicate was amplified in three independent reactions to avoid reaction-specific biases [57, 58]. To avoid contamination, each reaction was set up under a laminar flow cabinet, using only ultra-pure molecular-grade water (Sigma-Aldrich, Darmstadt, Germany) and a separate stock of reagents and plastics.

The reaction products were checked on a 1.5% agarose gel and purified using AMPure XP ratio of 1×. After that, all reactions were pooled, quantified using a Qbit instrument (Thermo Fisher Scientific, Waltham, Massachusetts, U.S.A.), and sequenced with 15% PhiX spike-in on a part of MiSeq sequencer lane (Illumina, San Diego, California, U.S.A.) using the 600-cycle MiSeq Reagent Kit v3 according to the manufacturer’s instructions.

#### NGS data analyses

The raw paired-end reads were merged using PEAR v. 0.9.8 [59] and only successfully merged reads were used in the next steps. Primers were then trimmed with Cutadapt v. 1.12 [60] and only reads containing primers were kept. Using vsearch v. 2.4.3 [61], the reads were filtered by the expected error rate (maxe) of 0.5, minimum length of 250 nt and maximum length of 450 nt, dereplicated, and the reads present in only one copy in a given individual were removed. Then, the reads were filtered using a custom Python script, retaining only the reads present in both replicates and in more than 100 copies in each replicate. The parameters were chosen after inspecting the sequences present in blank samples. After filtering, the reads from the duplicates were combined. The reads with 99% similarity were clustered using vsearch and the resulting sequences used in further analyses. The reads were also searched against NCBI database using BLASTn (https://blast.ncbi.nlm.nih.gov/Blast.cgi) to retain only the reads mapping against *Saxifraga*.

#### Phylogenetic reconstructions

In the first step, phylogenetic reconstructions with Maximum Likelihood (ML), Maximum Parsimony (MP) and Bayesian Inference (BI) methods were conducted for all three molecular marker regions separately and for both the extended and reduced datasets. ML searches and bootstrap estimation of clade support were conducted with RAxML 8.2.X [62]. This tool is provided under the familiar interface RAxML BlackBox with default settings on the CIPRES Science Gateway (www.phylo.org) [63]. On the same platform, we performed a Bayesian analysis employing MrBayes v. 3.1.2 [64] (rates = invgamma, ngen = 5,000,000, samplefreq = 500) to estimate the posterior probabilities (PP) of the Bayesian analyses. MP analyses with bootstrap calculations were performed using Paup* 4.0a152 [65] with the settings used by Tkach et al. [45]. Because the trees of the two plastid markers were identical, the respective sequence data were concatenated and analysed as one dataset. All trees were visualized with FigTree 1.4.3 (http://tree.bio.ed.ac.uk/software/figtree/). We used a bootstrap support (BS) ≥ 70% and PP ≥ 0.98 as thresholds to identify significant incongruence between clades of the plastid and the nrITS phylogenetic trees as previously suggested by Tkach et al. [45].

#### Molecular dating

The input file was produced with BEAUti, a tool of BEAST v. 1.8.4 [66], using default settings (see Additional file 2). We applied the uncorrelated lognormal clock model with Yule tree priors following Ebersbach et al. [46]. Four nodes were used as calibration points. The *Ribes* crown was constrained at 14.5 Ma and the *Ribes* stem node at minimally 48.9 Ma. We set the Iteaceae crown node constraint to 49 Ma and its stem node to 89 Ma according to Ebersbach et al. [46] and Gao et al. [47]. The node age prior distributions were always lognormal. Conservative settings (*m* = 1.5, SD = 1.0) were used to accommodate the degree of uncertainty. Four independent Markov chains were run for 100,000,000 generations and parameters were saved every 10,000th tree. Bayesian dating was performed in BEAST v. 1.8.4 [66]. The results were evaluated in Tracer v. 1.6 for effective sample size values and for determining the stationary phase of the runs [67]. Maximum clade credibility (MCC) trees were summarised in the BEAST tool TreeAnnotator v. 1.8.4 with 20% burn-in [66]. The final chronogram and its 95% highest posterior density (HDP) were visualised in FigTree 1.4.3.

## Results

### Sanger sequence data characteristics

The 44 new sequences of the *trn*L–*trn*F and ITS regions were added to the alignments used by Tkach et al. [45]. Structure and length of the alignments were not altered by the inclusion of these additional data. The alignment of the newly studied plastid region *rpl*32–*trn*L consisted of 248 sequences and had the length of 1117 base pairs (bp). The length of the *rpl*32–*trn*L sequences obtained varied from 434 bp in *S. hirculus* (sect. *Ciliatae*) to 747 bp in *S. funstonii* (sect. *Bronchiales*)*.* The considerable variation in sequence length (up to 313 bp) was caused by the presence of numerous long and partly nested structural mutations (indels).

### NGS sequencing output

Sequencing yielded 4,469,063 raw paired-end reads, including 7149 reads (0.16%) in blank samples, with an average of 58,709 reads per sample (excluding blank samples; standard deviation [SD]: 40,954; median: 66,261). Most of the paired-end reads were successfully merged for all the studied samples (mean: 94.5%; SD: 12.4%; median: 98.8%), and contained the targeted primer sequences (mean 98.1%; SD: 7.2%; median: 99.9% of merged reads). After quality filtering, a total of 3,110,076 reads were retained, including 5102 (0.16%) reads in blank samples (mean: 41,959; SD: 30,024; median: 45,346; excluding blank samples). No reads in blank samples were present in both technical replicates and in more than 100 copies, and reads from 29 of 38 samples passed the above filter. After clustering the reads with 99% similarity and discarding three sequences identified as fungal, an average of 1.9 ITS2 variants per sample (SD: 0.9; median: 2) was obtained, with the most abundant variant present 1–2 orders of magnitude more often than the less common ones. The 22 nrITS2 variants of *S. wahlenbergii* (11 samples), ten of *S. styriaca* (four samples), six of *S. androsacea* (three samples), three of *S. tridactylites* (two samples)*,* two each of *S. adscendens*, *S. depressa, S. seguieri* (one sample each), one variant each of *S. aphylla*, *S. discolor*, *S. italica, S. osloensis*, *S. praetermissa* (one sample each) were added to the alignment of the reduced dataset based on Sanger sequences.

### Molecular phylogenetics

#### Infrageneric placement of S. wahlenbergii and S. styriaca

**Fig. 3:**
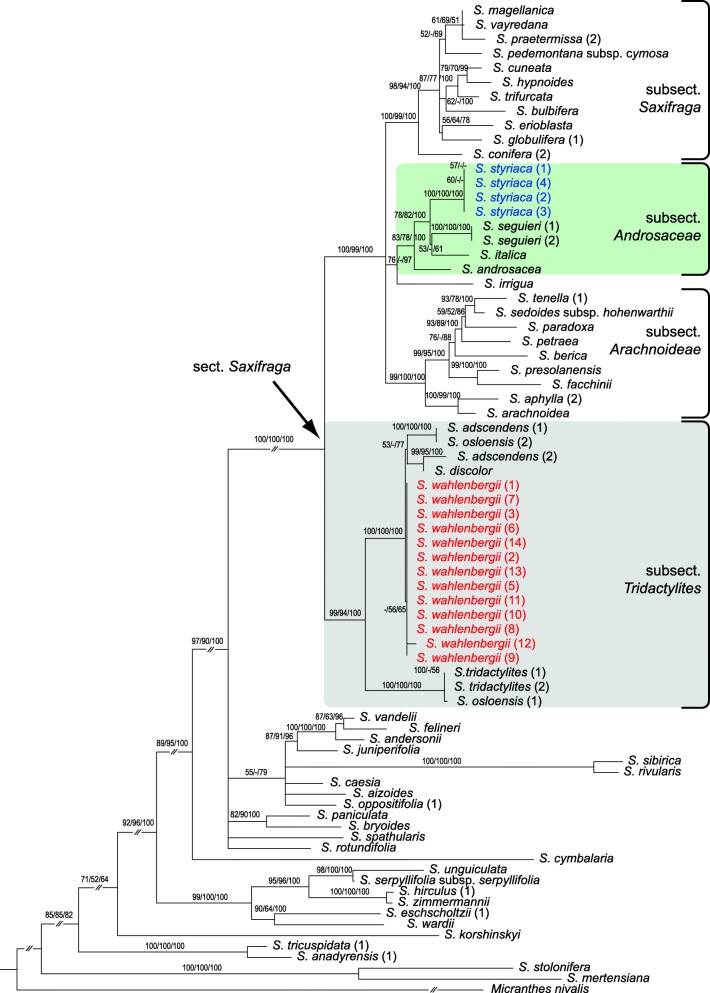
Maximum likelihood phylogenetic tree of *Saxifraga* sect. *Saxifraga* (arrow) and representatives of other sections of *Saxifraga* based on nrITS Sanger sequences (reduced dataset; see text for details). *Micranthes nivalis* was chosen as outgroup. Maximum likelihood and maximum parsimony bootstrap support values as well as posterior probabilities of Bayesian inference ≥50% are indicated on the branches. The subsections mentioned in the main text are labelled on the right-hand side

**Fig. 4:**
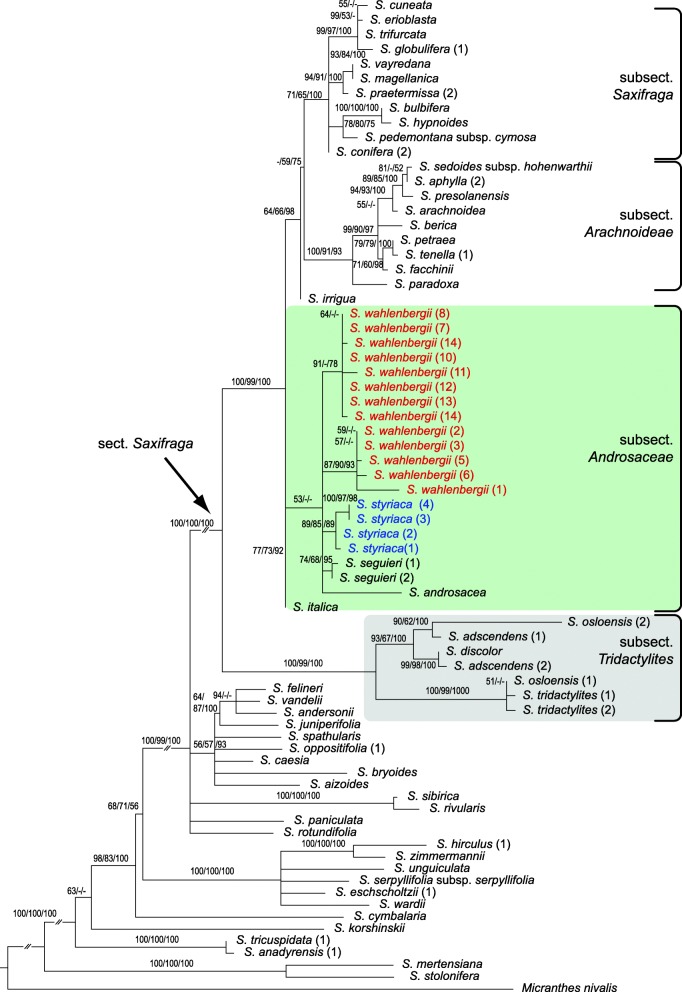
Maximum likelihood phylogenetic tree of *Saxifraga* sect. *Saxifraga* (arrow) and representatives of other sections of *Saxifraga* based on the plastid *trn*L–*trn*F and *rpl*32–*trn*L Sanger sequences (reduced dataset; see text for details). *Micranthes nivalis* was chosen as outgroup. Maximum likelihood and maximum parsimony bootstrap support values as well as posterior probabilities of Bayesian inference ≥50% are indicated on the branches. The subsections mentioned in the main text are labelled on the right-hand side

The topology, resolution and support of the individual trees based on the plastid *trn*L–*trn*F and *rpl*32–*trn*L regions were similar (not shown); therefore, both datasets were concatenated. The plastid and the nuclear ITS datasets for the entire genus *Saxifraga* (extended datasets) were analysed under ML, MP and BI. The tree topologies obtained for each dataset using each of the methods were widely corresponding, whereas resolution and branch support varied (see Additional file 3: Figure S1, Additional file 4: Figure S2). The plastid and nrDNA trees showed a congruent topology of major supported branches considering the threshold values of node support as outlined in the Material and methods section. In both trees, *S. wahlenbergii* and *S. styriaca* were firmly nested within the section *Saxifraga* (Additional file 3: Figure S1, Additional file 4: Figure S2), which also received strong support in the reduced datasets (nrITS: ML100/MP96/PP100, plastid: ML100/MP92/PP100; Figs. 3 and 4). Remarkably, this lineage is internally characterised by incongruent placement of several species, including *S. wahlenbergii*, among the nuclear and plastid DNA trees.

#### Phylogenetic affinities based on the reduced datasets

The reduced nrDNA tree based on Sanger sequences showed a basal dichotomy of the sect. *Saxifraga* lineage (Fig. 3). All accessions of *S. wahlenbergii* were included in the clade (ML99/MP94/PP100) containing the taxa belonging to subsect. *Tridactylites*: *S. adscendens, S. discolor*, *S. osloensis* and *S. tridactylites* (with accessions of *S. tridactylites* and one of *S. osloensis* forming a distinct branch within this clade). The second clade (ML100/MP99/PP100) encompassed all other species of sect. *Saxifraga*, which clustered in three well supported branches that corresponded to three other subsections: *Androsaceae*, *Arachnoideae* and *Saxifraga*. Accessions of *S. styriaca* grouped within the clade containing species from subsect. *Androsaceae* (*S. androsacea*, *S. italica*, *S. seguieri*). *S. irrigua* was included in a sister branch to the former group.

The plastid tree also revealed a basal split of section *Saxifraga* but with differences in the placement of some species (Fig. 4). In contrast to the ITS tree, the clade containing representatives of subsect. *Tridactylites* (ML100/MP99/PP100) did not contain *S. wahlenbergii* accessions. The sister clade (ML100/MP99/PP100) contained a dichotomy of the other species of this section, including a major subclade (ML77/MP73/PP92) with a polytomy encompassing the representatives of subsect. *Androsaceae* in accordance with the ITS tree: *S. androsacea*, *S. seguieri* and *S. styriaca*. Additionally, in contrast to the ITS tree, it contained all accessions of *S. wahlenbergii* grouped in two subclades. The second major subclade (ML64/MP66/PP98) contained the remaining species, namely *S. irrigua* (subsection placement uncertain), a clade grouping species of subsect. *Arachnoideae* (*S. arachnoidea*, *S. paradoxa*, etc.) and a clade grouping species of subsect. *Saxifraga* (*S. bulbifera*, *S. conifera*, *S. hypnoides*, *S. magellanica*, *S. praetermissa, S. vayredana*, etc.)

#### Analysis of ITS variation (Sanger and NGS data)

**Fig. 5:**
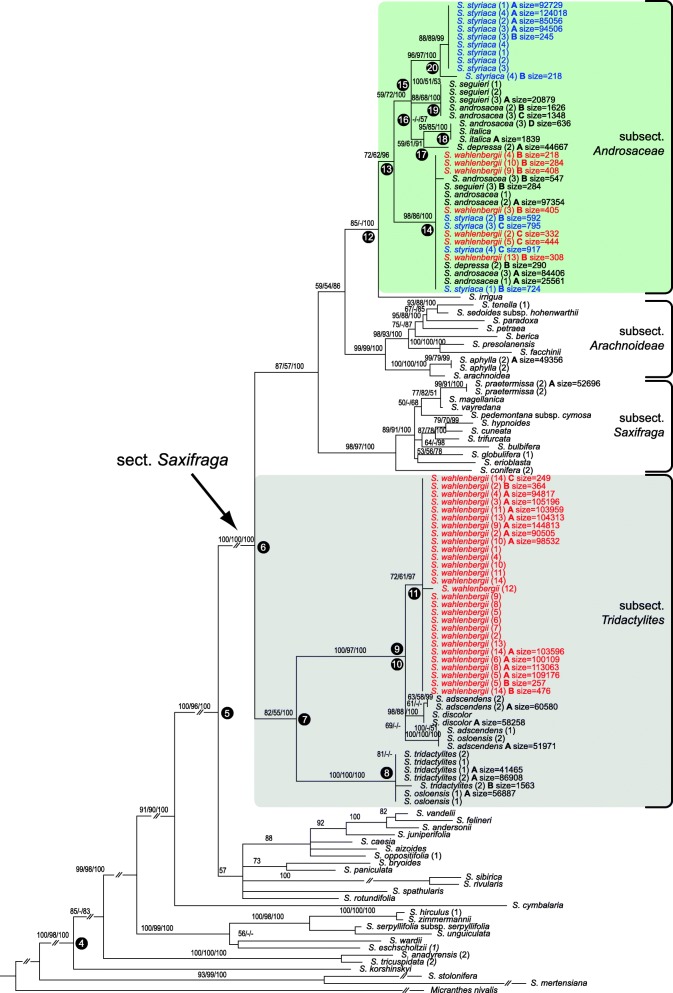
Maximum likelihood phylogenetic tree of *Saxifraga* sect. *Saxifraga* (arrow) and representatives of other sections of *Saxifraga* based on nrITS Sanger sequences and NGS reads (reduced dataset). *Micranthes nivalis* was chosen as outgroup. Maximum likelihood and maximum parsimony bootstrap support values as well as posterior probabilities of Bayesian inference ≥50% are indicated on the branches. The Arabic numerals behind taxon names indicate different provenances listed in Additional file 1: Table S1. Letters A–D mean different variants of NGS sequences, if present, followed by the number of NGS reads (size). Taxon names without such letter and without number of reads are Sanger sequences. Numbering of the main clades is according to Table 1. The subsections mentioned in the main text are labelled on the right-hand side. The clade 10 is not resolved

The NGS analysis of 28 samples of twelve species yielded 53 ITS2 variants. The tree based on Sanger ITS sequences and NGS data (Fig. 5) showed a similar topology as the reduced nrDNA dataset (Fig. 3). The majority of ITS2 variants of *S. wahlenbergii*, including all Sanger-based sequences, formed a coherent branch (node 10; ML72/MP61/PP97) in a clade comprising *S. adscendens*, *S. discolor* and *S. osloensis* (node 9; ML100/MP97/PP100). As in the reduced ITS dataset, the former clade was placed in a dichotomy with the one constituted by *S. tridactylites* and one accession of *S. osloensis* (node 8; ML100/MP100/PP100). Also in congruence with the phylogenetic tree based on the reduced ITS dataset (Fig. 3), most sequences of *S. styriaca* (Sanger and NGS) were placed in a single clade (node 17; ML96/MP97/PP100) with *S. androsacea, S. italica, S. seguieri* (all Sanger and NGS sequences) and *S. depressa* (NGS) as closest relatives (Fig. 5). Deviant phylogenetic relationships, not revealed by the Sanger sequencing analysis, were found for the remaining, less abundant, NGS variants of *S. wahlenbergii*. They were nested in a clade along with NGS variants of *S. androsacea, S. depressa, S. seguieri, S. styriaca* and the Sanger sequence of *S. androsacea* (node 12; ML98/MP86/PP100). This clade was represented in the Sanger-based phylogeny by *S. androsacea* (1) only (Fig. 3). In general, these minority NGS variants of *S. wahlenbergii* (obtained from seven out of eleven samples = 64%) and all sequences of *S. androsacea, S. depressa, S. italica, S. seguieri* and *S. styriaca* fell in one internally diversified clade (node 11; ML72/MP62/PP96) with *S. irrigua* as sister.

#### Divergence time estimation

**Fig. 6:**
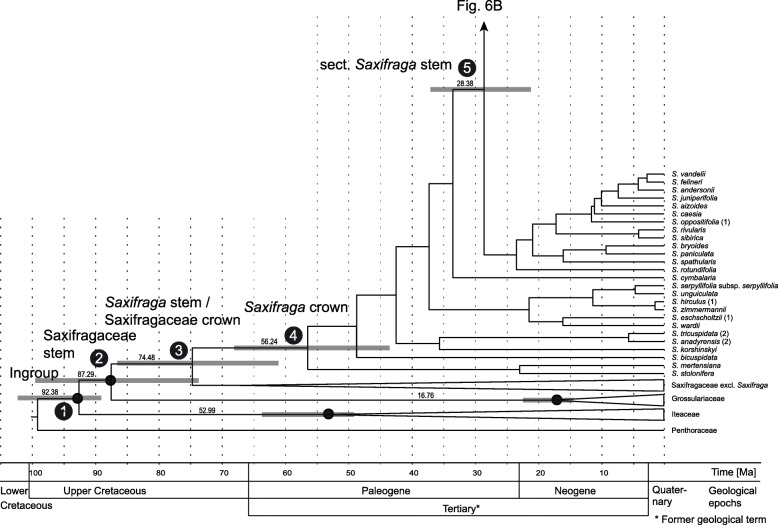

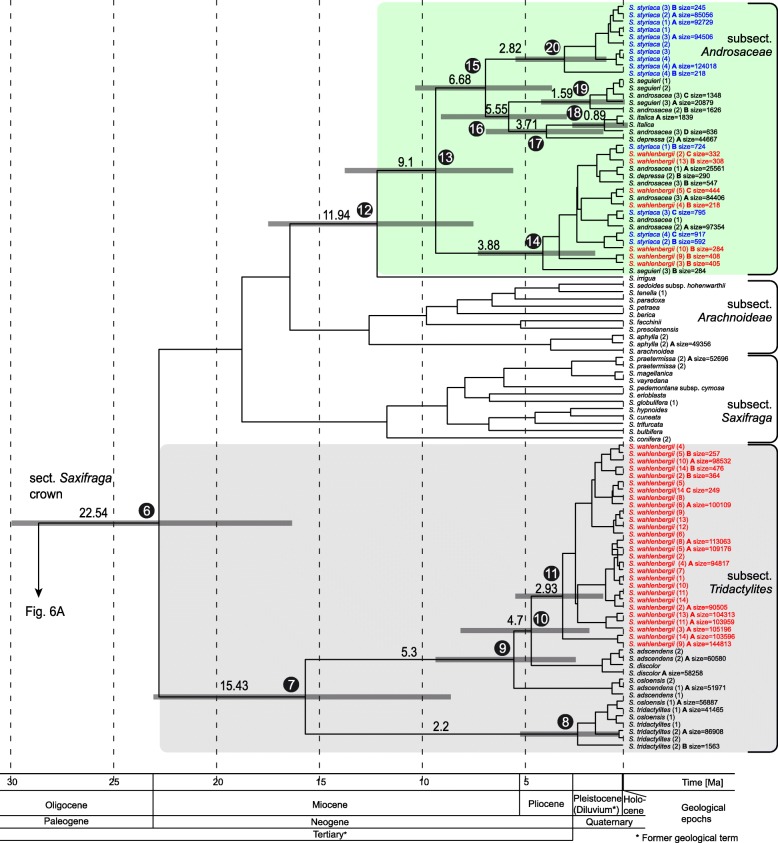
(**a** and **b**) Divergence time estimations for Saxifragaceae with focus on *Saxifraga* sect. *Saxifraga* (arrow) using an uncorrelated lognormal relaxed clock model applied for Sanger and NGS nrITS2 sequence data with the software BEAST v. 1.8.4. The numbers 1 to 17 refer to the main clades characterised in Table 1. Node heights indicate median ages. Horizontal node bars show the 95% posterior density probability date ranges. Four calibration points (see main text) are marked by circles. The time line with the main geological epochs is shown as horizontal axis in million years. Labelling behind taxon names as in Fig. 5

The divergence time estimations of relevant clades in *Saxifraga* based on the ITS dataset including the NGS reads of ITS2 are shown in Fig. 6. The origin of *Saxifraga* (stem) was estimated to 74.5 Ma (node 3; 95% HDP = 61.1–86.6) and the *Saxifraga* crown age to 56.1 Ma (node 4; 95% HDP = 43.6–68.1). The stem age of *Saxifraga* sect. *Saxifraga* was estimated to 28.3 Ma (node 5; 95% HDP = 21.2–37.1). These age values are comparable to the results of Ebersbach et al. [46] and Gao et al. [47].

Stem ages of *Saxifraga* subsect. *Tridactylites* and subsect. *Androsaceae* were dated to 22.6 Ma (node 6; 95% HDP = 16.3–29.9) and 12.0 Ma (node 11; 95% HDP = 7.5–17.5), respectively. The crown age of subsect. *Tridactylites* was dated to 15.5 Ma (node 7; 95% HDP = 8.6–23.1), when the lowland species *S. tridactylites* and one accession of *S. osloensis* (1) segregated from the alpine species group of *S. adscendens*, *S. discolor*, *S. wahlenbergii* and one accession of *S. osloensis* (2). *Saxifraga wahlenbergii* (*Tridactylites*-related sequence) segregated from *S. adscendens* at ca. 4.7 Ma (node 10; 95% HDP = 2.4–8.9).

Diversification within the subsect. *Androsaceae* occurred after 9.1 Ma (node 12; 95% HDP = 5.6–13.8; crown age). The widespread *S. androsacea* seems to be the oldest species in this section (Fig. 6). *Saxifraga styriaca* split off the other species from the sister clade to *S. androsacea* at approximately 6.7 Ma (node 14; 95% HDP = 3.7–10.3).

## Discussion

### Phylogenetic position of *Saxifraga wahlenbergii* and *S. styriaca*

According to the nuclear and plastid DNA data, the Central European narrow mountain endemics *S. wahlenbergii* (Western Carpathians) and *S. styriaca* (Eastern Alps) are firmly nested within the large *S.* sect. *Saxifraga* (Figs. 3 and 4; Additional file 3: Figure S1, Additional file 4: Figure S2). This group is one of ca. 13 sections of the genus *Saxifraga* [45] and accommodates altogether 70–85 species (about 16% of the total species number) with predominantly European distribution. The phylogenetic placement based on 14 *S. wahlenbergii* and four *S. styriaca* samples used in this study corroborates previous preliminary reports [44, 45].

Within *S.* sect. *Saxifraga*, the inferred phylogenetic relationships of *S. wahlenbergii* and *S. styriaca* are different for the nuclear and plastid data. In the ITS tree based on Sanger sequences, they are placed in two different main subclades (Fig. 3), which does not support their status as sister species hypothesized based on morphological features [22]. In contrast, both species are indeed closely related based on the plastid DNA tree where they belong to the same clade together with several species of *S.* subsect. *Androsaceae* (Fig. 4). While the affinity to this subsection is congruently displayed by *S. styriaca* in the two datasets*, S. wahlenbergii* has a nrDNA resembling that of *S. adscendens*, *S. discolor*, *S. osloensis* and *S. tridactylites* (subsect. *Tridactylites*) and plastid DNA resembling that of *S. androsacea*, *S. italica* and *S. seguieri* (subsect. *Androsaceae*). This incongruence suggests that *S. wahlenbergii* originated from hybridisation between representatives of these subsections, supporting the hypothesis of Tkach et al. [45], which was, however, based on a single sample of this species.

Incidentally, it is worth noting that *S. osloensis,* a tetraploid species originating from hybridisation between *S. adscendens* and *S. tridactylites* [45, 68], is represented in this study by different maternal ITS DNA copies as revealed by comparison of nuclear and plastid DNA trees (Figs. 3 and 4), namely the one of *S. tridactylites* in accession *S. osloensis* (1) and the one of *S. adscendens* in accession *S. osloensis* (2). For the accession *S. osloensis* (1) used in this study, the second parental ITS sequence was detected neither in Sanger analysis nor in NGS reads (Fig. 5). The sequence data for *S. osloensis* (2) were taken from GenBank (Sanger only; see Additional file 1: Table S1). A recurrent origin of this hybrid taxon is likely to explain placement of its accessions in different subclades of our tree.

### NGS data provide evidence for introgression

The NGS screening of nuclear ITS2 proved efficient in retrieving within-individual sequence variation and detecting both majority and minority variants present in genomes. It shed further light on the origin of the focal species and provides information not retrieved from the Sanger sequencing. The by far predominant number of the sequence reads (99.8%) in *S. wahlenbergii* was placed in the lineage comprising the species of *S.* subsect. *Tridactylites*, as expected from the Sanger sequencing data (Fig. 5). Here, *S. wahlenbergii* sequences (Sanger and NGS) formed a subclade most closely related to *S. adscendens*, *S. discolor* and one accession of *S. osloensis*. However, the NGS analysis additionally unravelled a minority set of sequences in *S. wahlenbergii*, which was placed within the subclade of *S*. subsect. *Androsaceae*, thus corroborating the plastid tree. These sequences were far less numerous (0.2%) and were detected in most but not all individuals (64%). They grouped with the majority of *S. androsacea* reads (98.2%) from various geographical regions and some minority ITS2 variants of *S. depressa, S. seguieri, S. styriaca*. Minority variants detected in the last-mentioned three species may have arisen via hybridization or introgression from *S. androsacea* (see below) and the vast majority of their NGS reads (99% in the case of *S. styriaca*) were placed outside this clade and followed the topology detected in the Sanger-based phylogeny (Fig. 3). The presence of minority ITS2 variants incongruent with the placement of major variants could arguably be suspected as possible sample contamination, but we took a special care, both during the lab procedures and in filtering the obtained reads, keeping only those present in more than 100 copies in both independent reaction replicates.

A minority of the NGS variants in *S. depressa* and *S. styriaca* (0.1%) grouped together and mixed up with the Sanger sequence of *S. androsacea* and NGS variants of *S. androsacea, S. seguieri* and *S. wahlenbergii* (ML98/MP86/PP100; Fig. 5).

In general, in the complex pattern of genetic introgression observed between the species constituting the clade of subsect. *Androsaceae* (Fig. 5), three scenarios were observed. (1) Within *S. depressa*, *S. styriaca* and *S. wahlenbergii* minority ITS variants of *S. androsacea* were found but not vice versa. (2) Between *S. seguieri* and *S. androsacea*, there was reciprocal exchange. (3) Within *S. androsacea* a copy of nrITS of *S. italica* was found but not vice versa. This evidence concurs with frequent hybridisation reported for *Saxifraga androsacea* throughout its range, essentially with species placed in the same subsection. Described hybrids include *S. androsacea × S. styriaca* = *S*. ×*melzeri* Köckinger, *S. androsacea* × *S. depressa* = *S.* ×*vierhapperi* Handel-Mazetti, *S. androsacea* × *S. seguieri* = *S*. ×*padellae* Brügger, *S. androsacea* × *S. wahlenbergii* = *S*. ×*thrinax* Rechinger (the parental taxa of the latter hybrid however are considered uncertain; e.g., [13]).

The extensive hybridisation within subsect. *Androsaceae* seems to reflect the geographical distribution of these high-mountain plants. *Saxifraga androsacea* is the most widespread and its distribution encompasses wide parts of Eurasia including mountains of Europe, the eastern Altai, mountains west of Lake Baikal and the Himalayas of Pakistan. It overlaps with the ranges of the narrowly distributed endemics *S. depressa* (Eastern Alps: Dolomites)*, S. seguieri* (middle part of the Alpine arc)*, S. styriaca* (Eastern Alps: Lower Tauern)*, S. wahlenbergii* (Western Carpathians). No hybrids between *S. androsacea* and *S. italica* were reported, which obviously agrees with the absence of *S. androsacea* in the Central Apennines, where *S. italica* occurs*.* However, an NGS-based ITS copy congruent with *S. italica* was observed in the Pyrenean accession of *S. androsacea* in our data set. It is worth noting that the DNA from this *S. androsacea* sample was isolated in a different laboratory than the sample of *S. itali*ca. Therefore, cross-contamination is an unlikely explanation of this pattern.

*Saxifraga androsacea* is cytogenetically highly variable; chromosome numbers of 2*n* = 16 (likely erroneous), 66, 88, 105, ca. 112, ca. 120, 124, 128, 154, 192, 198, 206–220, 208, 210, 220 were reported (Chromosome Counts Database [CCDB], http://ccdb.tau.ac.il/ [69]). The count of 2*n* = 220 represents the highest chromosome number in *Saxifraga* [70]. The wide range of chromosome numbers points to an important role of polyploid evolution in *S. androsacea* and, indeed, allopolyploidy could explain the presence of several different ITS variants found in this species. Chromosome numbers of other species (see [69]) are rather uniform and clearly based on *x* = 11, namely *S. italica* (2*n* = 66), *S. seguieri* (2*n* = 66), *S. wahlenbergii* (2*n* = 66); no counts are available for *S. depressa* and *S. styriaca*. In the case of *S*. subsect. *Tridactylites* – involved in formation of *S. wahlenbergii* – disregarding the geographically remote, tetraploid *S. osloensis* (2*n* = 44), both *S. adscendens* and *S. tridactylites* are diploids with 2*n* = 22 chromosomes.

### Morphological similarity of *S. wahlenbergii* and *S. styriaca*

**Fig. 7:**
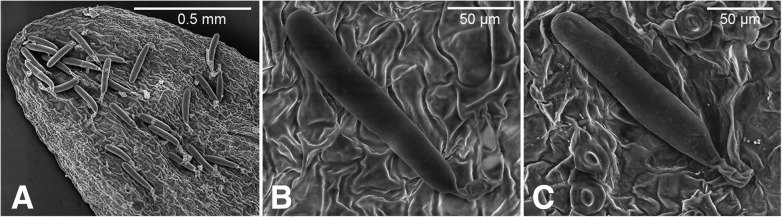
SEM photomicrographs of leaf trichomes in *Saxifraga wahlenbergii* (**a**, **b**) and *S. styriaca* (**c**). See Methods for the specimens studied

*Saxifraga wahlenbergii* has previously been treated as a separate, monotypic ‘grex’ or series *Perdurantes* (S.Pawł.) S.Pawł. [18, 71]. The main diagnostic character of *Perdurantes* was the presence of peculiarly shaped glandular hairs that were later found also in *S. styriaca* ([22]; Fig. 7) but are absent in all other species of *Saxifraga* studied so far [72]. This character induced Köckinger [22] to suggest an intimate relationship between *S. styriaca* and *S. wahlenbergii*, which also share characters of the leaf morphology, protogynous flowering and a similar growth form (Fig. 1). Köckinger [22], however, also noted considerable differences at flowering time when both species are unmistakable. The inflorescences are consistently one-flowered in *S. styriaca* versus usually several-flowered in *S*. *wahlenbergii*, petals are minute and yellowish-green in *S. styriaca* whereas they are large and white in *S. wahlenbergii* (Fig. 1a–c). According to our results, the peculiar glandular hairs of *S. styriaca* and *S. wahlenbergii* (Fig. 7) are seemingly a homoplasious character and do not represent a synapomorphy of both species but rather have a parallel, independent origin. Alternatively, but less likely, one might assume an extinct *S. androsacea*-like ancestor with this type of hairs currently found only in *S. wahlenbergii* and *S. styriaca*.

The close relationship of *S. wahlenbergii* and the Pyrenean-Cantabrian *S. praetermissa*, suggested by Engler [73], Engler & Irmscher [74] and Webb & Gornall [13], who placed both species in a separate series *Axilliflorae* (Willk.) Pawł., was not supported by our molecular phylogenetic results. *Saxifraga praetermissa* was placed within a clade containing other species from subsect. *Saxifraga*, distant to *S. wahlenbergii* in both plastid and nuclear ITS trees (Figs. 3, 4 and 5). This agrees with differences in the position of the flowering stems (axillary in *S. praetermissa* and terminal in *S. wahlenbergii*) as documented by Pawłowska [18], despite overall similar appearance of both species. In the same line, the suggestion by Pax [14] that *S. wahlenbergii* is related to *S. moschata* was not supported by phylogenetic data. It is important to note that the relationships of *S. wahlenbergii* found in our study were already considered by Ball [75] in the original description of *S. wahlenbergii*. He clearly pointed to its similarities to *S. androsacea* as well as to *S. adscendens* (as *S. controversa* Sternb.) in the case of specimens growing at lower elevations.

*Saxifraga styriaca*, on the other hand, was initially misidentified as *S. aphylla*, a more widespread endemic species of the Eastern Alps because of similar 3-lobed cuneate leaves, leafless flowering stems, narrow and short petals [22]. The decumbent stems, later flowering time, protandry, petals longer than sepals, shortly stalked hairs with spherical heads and ecological characteristics of *S. aphylla*, however, are in contrast to the typical features of *S. styriaca*. These morphological differences were underscored by the molecular phylogenetic placement of *S. aphylla* distant to *S. styriaca* in a clade representing subsect. *Arachnoideae* (Engl. & Irmscher) Tkach, Röser & M.H.Hoffm. (Figs. 3, 4 and 5).

### Hybrid origin of *S. wahlenbergii*

The combination of plastid and ITS sequences indicates that the paternal ancestor of *S. wahlenbergii* was a species closely related to *S. adscendens* (Figs. 3 and 5), if not *S. adscendens* itself. In contrast to the majority of saxifrages, all species of subsect. *Tridactylites* are short-lived. *Saxifraga tridactylites* and *S. osloensis* are annual, whereas *S. adscendens* and *S. discolor* are biennial. It can be assumed that the maternal parent contributing the subsect. *Androsaceae*-like plastid DNA was perennial, as this is the only life form present in this subsection, and this life form was established in *S. wahlenbergii*. Nuclear and plastid DNA do not provide an unequivocal indication as to which species of subsect. *Androsaceae* was the actual parent of *S. wahlenbergii*. Considering the biogeographical context, it was most likely *S. androsacea*, because it is currently a widespread species and the only representative of this subsection that inhabits the Western Carpathians. This is further corroborated by the placement of subsect. *Androsaceae*-like NGS ITS variants of *S. wahlenbergii* within the clade of *S. androsacea*.

The occurrence of a significantly lower number of NGS reads of subsect. *Androsaceae*-like nuclear ITS in *S. wahlenbergii* (Fig. 5) may suggest that the maternal ITS was mostly replaced by subsect. *Tridactylites-*like variants following the hybridisation. In four of eleven samples of *S. wahlenbergii* analysed with NGS subsect. *Androsaceae*-like variants could not be detected (*S. wahlenbergii* 6, 8, 11, 14; Fig. 5). Such unidirectional loss through the process of concerted evolution, involving gene conversion and recombination, is frequent in the repetitive ribosomal DNA [76–81]. Alternatively, the presence of subsect. *Androsaceae*-like ITS variants in *S. wahlenbergii* may result from infrequent cases of more recent introgression. Based on our data it is not possible to discern between these two explanations; also a combination of both processes cannot be ruled out. In any event, hybridisation is clearly witnessed by the exclusive presence of plastid DNA from the *S. androsacea* lineage in all accessions of *S. wahlenbergii*.

The presumable paternal parent, *S. adscendens*, is disjunctly distributed in Fennoscandia, the European Alpine System, Anatolia and the Caucasus as well as in the mountains of western North America. In the Western Carpathians, its distribution overlaps with the range of *S. androsacea*, which makes it likely that *S. wahlenbergii* originated in situ in this area. The other species of subsect. *Tridactylites* nested in the clade of *S. wahlenbergii* and *S. adscendens* (Figs. 3 and 5) occupy narrow ranges distant to the Western Carpathians. *Saxifraga discolor* (syn. *S. adscendens* var. *discolor*) occurs in the Balkan Peninsula [13], while the hybrid species *S. osloensis* occurs only in a small area of southern Scandinavia [82]. The predominantly lowland plant *S. tridactylites*, which is also widespread in Europe, can be excluded as parent of *S. wahlenbergii* due to its distant position in the phylogenetic trees (Figs. 3, 4 and 5).

### Temporal frame

According to our divergence time estimation, the maximal age of the origin of *S. wahlenbergii* is set later than 4.7 Ma, when its paternal, *S. adscendens*-like ITS DNA originated (Fig. 6). The hybridisation with the second parent representing subsect. *Androsaceae*, occurred necessarily at a later time. It could correspond to the late Pliocene cooling periods or the Pleistocene. The earliest glaciation in Poland is now dated to the uppermost Lower Pleistocene (about 0.9 Ma) when the ice sheet reached the mid-southern part of the country [83]. Even postglacial origin of *S. wahlenbergii* may be considered, because *S. adscendens* and *S. androsacea* presently co-exist in the Tatra Mountains and neighbouring massifs, partly in a similar altitudinal range [84, 85]. However, an older origin of the species seems more likely for several reasons. First, the origin of *S. wahlenbergii* involves two remotely related taxa for which no contemporary hybridisation has been reported to date [13]. Thus, a hybridisation incident could be expected earlier in their evolutionary history and maybe also in significantly different ecological conditions than those acting today [86]. Second, the subsect. *Tridactylites-*related sequence of *S. wahlenbergii* shows some divergence from sequence of the extant *S. adscendens* lineage (Fig. 5; checked also in additional specimens of *S. adscendens* growing in sympatry with *S. wahlenbergii*; E. Cieślak, M. Ronikier, unpubl. data). Finally, some degree of geographically-driven intraspecific diversification observed in *S. wahlenbergii* (Fig. 4; E. Cieślak & M. Ronikier, unpubl. data) also suggests a longer than postglacial historical context for this taxon.

*Saxifraga wahlenbergii* was usually considered an ‘ancient’ Tertiary relict of the Western Carpathian flora [17–19, 87], even though it was not explicitly implied that it belonged to the circumboreal flora that thrived under warm and wet climatic conditions during the early to mid-Tertiary (65–15 Ma) [88, 89]. Pawłowski [17] suggested an origin of *S. wahlenbergii* in the ‘pre-Diluvial’ period and mentioned a time span from Pliocene to Early Pleistocene. Our results on the maximum potential age of this endemic species agree with Pawłowski’s estimation and certainly speak against an origin of *S. wahlenbergii* during the Tertiary climatic optimum. The Pleistocene glaciations and related importance of stable refuge areas are among important historical events that increase diversity of (endemic) vascular plants as reviewed by Bruchmann & Hobohm [7]. On the other hand, distribution shifts induced by recurrent climatic changes may be particularly important for hybrid speciation.

Dated phylogenies for the Carpathian endemic plants species are still scarce [3]. A recent study on *Syringa josikea*, an endemic shrub of temperate montane forests in the Apuseni Mountains and the Eastern Carpathians, established an age of ca. 1.9 Ma (Early Pleistocene) for the segregation from its closest relatives [90]. Forest species like *Syringa josikea* have presumably survived the Last Glacial Maximum (LGM) in lowlands surrounding the Carpathians or even within the mountain range [90]. It can be expected that *S. wahlenbergii*, a plant reaching alpine and subnival zones, may have survived the (maximally) past 4.7 Ma, a period spanning the entire Pleistocene, in situ in the Western Carpathians by following the shifts of the altitudinal vegetation caused by the glacial-interglacial cycles as discussed for several other alpine species in the region (e.g., [43, 91]). Such vertical migrations are important for in situ survival of alpine plant species. The cold periods have even led to an extensive increase of suitable habitats for alpine plants in lower altitudes of the Carpathians ([2]: Fig. 2). On the other hand, glacial survival within the Western Carpathians is considered likely also for species of the montane woodland zone [92] suggesting maintenance of a wide range of ecological conditions.

The origin of *Saxifraga styriaca* (ca. 6.8 Ma) predates the Pleistocene and reaches back to the Late Miocene (Messinian), overlapping with the final phase of the Alpine orogeny that lasted until 2.6 Ma [93]. *S. styriaca*, with a narrow range covering only ca. 1250 km^2^, agrees with a typical pattern of an Alpine endemic plant species as it belongs to a lineage otherwise widespread in the European Alpine System [11]. It likely belongs to the elements of the Alpine flora that survived the Pleistocene glaciations in the refuge areas in situ. Its present-day distribution fits largely the most important glacial refuge area for silicicolous plants in the Eastern Alps (region S1 in [94]: Fig. 5; [95, 96]).

## Conclusions

The evolutionary history of the Eastern Alpine endemic *S. styriaca* is strongly different from that of West Carpathian *S. wahlenbergii*. They are neither sister species that would provide an example of vicariance or dispersal nor do they represent a link between the floras of the Eastern Alps and the Carpathians. *Saxifraga styriaca* seems to have been even more conservative than the Carpathian endemic in keeping its very restricted present-day range within the refuge area. Our study underlines the importance of multilocus DNA studies in endemic species along with their close relatives to clarify the evolutionary and biogeographical connections between the floras of high mountain regions.

## Additional files

Additional file 1: **Table S1.** Taxa included in the phylogenetic analysis. ENA/GenBank accession numbers are given for all ITS, *trn*L–*trn*F and *rpl*32–*trn*L Sanger sequences used in this study, followed by available ITS2 sequences obtained from NGS (without ENA/GenBank entry). They are denoted as “ITS2 variants A–D”. Missing sequence data are indicated by “NA” (not available). Voucher data (country and region, collector and collection number, herbarium code according to Thiers et al. [97]) are provided for newly generated sequences, which are in bold print. MR: herbarium of G. & S. Miehe deposited at the Institute of Geography, University Marburg, Germany. (PDF 502 kb)

Additional file 2: Sequence alignments (FASTA) used in this study (ITS extended taxon set, ITS reduced taxon set, ITS reduced taxon set with NGS reads, ITS reduced taxon set with NGS reads for molecular clock, *trn*L–*trn*F and *rpl*32–*trn*L extended taxon set, *trn*L–*trn*F and *rpl*32–*trn*L reduced taxon set) and the BEAUti-generated XML file. See Methods for details and explanation. (ZIP 189 kb)

Additional file 3: **Figure S1.** Maximum likelihood phylogram of *Saxifraga* (arrow) and representative genera of Saxifragaceae based on nuclear ribosomal ITS DNA Sanger sequence data. *Pterostemon rotundifolius* and *Itea virginica* (Iteaceae) were chosen as outgroups. Maximum likelihood and maximum parsimony bootstrap support values as well as posterior probabilities of Bayesian inference ≥50% are indicated on the branches. The sections of *Saxifraga* are labelled on the right-hand side. (PDF 2680 kb)

Additional file 4: **Figure S2.** Maximum likelihood phylogram of *Saxifraga* (arrow) and representative genera of Saxifragaceae based on plastid *trn*L–*trn*F and *rpl*32–*trn*L Sanger sequence data. *Pterostemon rotundifolius* and *Itea virginica* (Iteaceae) were chosen as outgroups. Maximum likelihood and maximum parsimony bootstrap support values as well posterior probabilities of Bayesian inference ≥50% are indicated on the branches. The sections of *Saxifraga* are labelled on the right-hand side. (PDF 793 kb)

## References

[1] Ozenda P (1985). La végétation de la chaîne alpine dans l’espace montagnard européen.

[2] Ronikier M (2011). Biogeography of high-mountain plants in the Carpathians: an emerging phylogeographical perspective. Taxon.

[3] Mráz P, Ronikier M (2016). Biogeography of the Carpathians: evolutionary and spatial facets of biodiversity. Biol J Linn Soc.

[4] Mráz P, Barabas D, Lengyelová L, Turis P, Schmotzer A, Janišová M, Ronikier M (2016). Vascular plant endemism in the Western Carpathians: spatial patterns, environmental correlates and taxon traits. Biol J Linn Soc.

[5] Tzedakis PC, Emerson BC, Hewitt GM (2013). Cryptic or mystic? Glacial tree refugia in northern Europe. Trends Ecol Evol.

[6] Schmitt T, Catalan J, Ninot J, Aniz M (2017). Molecular biogeography of the high mountain systems of Europe: an overview. High mountain conservation in a changing world. Advances in global change research.

[7] Bruchmann I, Hobohm C, Hobohm C (2014). Factors that create and increase endemism. Endemism in vascular plants.

[8] Pawłowski B (1970). Remarques sur l'endémisme dans la flore des Alpes et des Carpates. Vegetatio.

[9] Hurdu B-I, Escalante T, Puşcaş M, Novikoff A, Bartha L, Zimmermann NE (2016). Exploring the different facets of plant endemism in the south-eastern Carpathians: a manifold approach for the determination of biotic elements, centres and areas of endemism. Bot J Linn Soc.

[10] Aeschimann D, Rasolofo N, Theurillat J-P. Analyse de la flore des Alpes, vol. 1: Historique et biodiversité. Candollea. 2011a;66:27–55.

[11] Kadereit JW. The role of *in situ* species diversification for the evolution of high vascular plant species diversity in the European Alps. – A review and interpretation of phylogenetic studies of the endemic flora of the Alps. Perspect Pl Ecol Evol Syst. 2017;26:28–38.

[12] Aeschimann D, Rasolofo N, Theurillat J-P (2011). Analyse de la flore des Alpes, vol. 2: Biodiversité et chorologie. Candollea.

[13] Webb DA, Gornall RJ (1989). Saxifrages of Europe: with notes on African, American and some Asiatic species.

[14] Pax F. Grundzüge der Pflanzenverbreitung in der Karpathen, vol. 1–2. Leipzig: Engelmann; 1898–908.

[15] Kliment J (1999). Komentovaný prehľad vyšších rastlín flóry Slovenska, uvádzaných v literatúre ako endemické taxóny [Annotated survey of the vascular plants of the Slovak flora recorded in the literature as endemic taxa]. Bull Slov Bot Společnost.

[16] Engler HGA, Irmscher E. Saxifragaceae – *Saxifraga* II. In: Engler HGA, editor. Pflanzenreich, vol. 69 (IV, 117), Saxifragaceae – *Saxifraga*. Leipzig: Engelmann; 1919. p. [1–47] 449–709.

[17] Pawłowski B (1928). Die geographischen Elemente und die Herkunft der Flora der subnivalen Vegetationsstufe im Tatra-Gebirge. Bull Int Acad Polon Sci, Cl Sci Math, Sér B, Sci Nat.

[18] Pawłowska S. De positione systematica speciei *Saxifraga Wahlenbergii* Ball (= *S*. *perdurans* Kit.). Fragm Florist Geobot. 1966;12:337–47.

[19] Skalińska M (1963). Cytological studies in the flora of the Tatra Mts. A synthetic review. Acta biol Cracov. Ser Bot.

[20] Schneeweiss GM, Schönswetter P (1999). Feinverbreitung, Ökologie und Gesellschaftsanschluß reliktischer Gefäßpflanzen der östlichen Niederen Tauern (Steiermark, Österreich). Stapfia.

[21] Tribsch A (2004). Areas of endemism of vascular plants in the eastern Alps in relation to Pleistocene glaciation. J Biogeogr.

[22] Köckinger H (2003). *Saxifraga styriaca* spec. Nova (Saxifragaceae) – an endemic species of the eastern Niedere Tauern Mts. (Styria, Austria). Phyton.

[23] Piękoś-Mirkowa H, Mirek Z, Miechówka A (1996). Endemic vascular plants in the polish Tatra Mts. – distribution and ecology. Polish Bot Stud.

[24] Pawłowski B (1956). Flora Tatr: Rośliny naczyniowe.

[25] Caudullo G, de Rigo D, San-Miguel-Ayanz J, de Rigo D, Caudullo G, Houston Durrant T, Mauri A (2016). *Pinus cembra* in Europe: distribution, habitat, usage and threats. European atlas of forest tree species.

[26] Höhn M, Gugerli F, Abran P, Bisztray G, Buonamici A, Cseke K, Hufnagel L, Quintela-Sabarís C, Sebastiani F, Vendramin GG (2009). Variation in the chloroplast DNA of Swiss stone pine (*Pinus cembra* L.) reflects contrasting post-glacial history of populations from the Carpathians and the Alps. J Biogeogr.

[27] Mráz P, Gaudeul M, Rioux D, Gielly L, Choler P, Taberlet P, IntraBioDiv Consortium (2007). Genetic structure of *Hypochaeris uniflora* (Asteraceae) suggests vicariance in the Carpathians and rapid post-glacial colonization of the Alps from an eastern alpine refugium. J Biogeogr.

[28] Koch M, Dobeš C, Bernhardt KG, Kochjarová J (2003). *Cochlearia macrorrhiza* (Brassicaceae): a bridging species between *Cochlearia* taxa from the eastern Alps and the Carpathians?. Plant Syst Evol.

[29] Klein JT, Kadereit JW (2015). Phylogeny, biogeography, and evolution of edaphic association in the European orophytes *Sempervivum* and *Jovibarba* (Crassulaceae). Int J Pl Sci.

[30] Zhang L-B, Comes HP, Kadereit JW (2001). Phylogeny and quaternary history of the European montane/alpine endemic *Soldanella* (Primulaceae) based on ITS and AFLP variation. Amer J Bot..

[31] Bellino A, Bellino L, Baldantoni D, Saracino A (2015). Evolution, ecology and systematics of *Soldanella* (Primulaceae) in the southern Apennines (Italy). BMC Evol Biol.

[32] Meusel H, Mühlberg H, Hegi G (1979). Unterfamilie Silenoideae (Lindl.) A.Br. Illustrierte Flora von Mitteleuropa, vol. III/2.

[33] Aeschimann D, Lauber K, Moser DM, Theurillat J-P (2004). Flora alpina.

[34] Ronikier M, Cieślak E, Korbecka G (2008). High genetic differentiation in the alpine plant *Campanula alpina* Jacq. (Campanulaceae): evidence for glacial survival in several Carpathian regions and long-term isolation between the Carpathians and the Alps. Molec Ecol.

[35] Pachschwöll C, Escobar García P, Winkler M, Schneeweiss GM, Schönswetter P (2015). Polyploidisation and geographic differentiation drive diversification in a European high mountain plant group (*Doronicum clusii* aggregate, Asteraceae). PLoS One.

[36] Pachschwöll C, Pușcaș M, Schönswetter P (2011). Distribution of *Doronicum clusii* and *D. stiriacum* (Asteraceae) in the Alps and Carpathians. Biologia (Bratislava).

[37] Puşcaş M, Choler P (2012). A biogeographic delineation of the European alpine system based on a cluster analysis of *Carex curvula*-dominated grasslands. Flora.

[38] Kropf M, Kadereit JW, Comes HP (2003). Differential cycles of range contraction and expansion in European high mountain plants during the Late Quaternary: insights from *Pritzelago alpina* (L.) O. Kuntze (Brassicaceae). Mol Ecol.

[39] Paun O, Schönswetter P, Winkler M, Tribsch A (2008). Evolutionary history of the *Ranunculus alpestris* group (Ranunculaceae) in the European Alps and the Carpathians. Molec Ecol..

[40] Alsos I, Alm T, Normand S, Brochmann C (2009). Past and future range shifts and loss of diversity in dwarf willow (*Salix herbacea* L.) inferred from genetics, fossils and modelling. Glob Ecol Biogeogr.

[41] Ronikier M, Schneeweiss GM, Schönswetter P (2012). The extreme disjunction between Beringia and Europe in *Ranunculus glacialis* s. l. (Ranunculaceae) does not coincide with the deepest genetic split – a story of the importance of temperate mountain ranges in arctic-alpine phylogeography. Molec Ecol..

[42] Schmickl R, Paule J, Klein J, Marhold K, Koch MA (2012). The evolutionary history of the *Arabidopsis arenosa* complex: diverse tetraploids mask the Western Carpathian center of species and genetic diversity. PLoS One.

[43] György Z, Vouillamoz J, Höhn M (2016). Microsatellite markers reveal common east alpine-Carpathian gene pool for the arctic-alpine *Rhodiola rosea* (Crassulaceae). Pl Syst Evol.

[44] Cieślak E, Ronikier M, Schönswetter P (2013). Phylogenetic analysis confirms the status of *Saxifraga wahlenbergii* Ball (Saxifragaceae) as a distinct endemic of the Western Carpathians. Acta Biol Cracov, Ser. Bot.

[45] Tkach N, Röser M, Miehe G, Muellner-Riehl AN, Ebersbach J, Favre A, Hoffmann MH (2015). Molecular phylogenetics, morphology and a revised classification of the complex genus *Saxifraga* (Saxifragaceae). Taxon.

[46] Ebersbach J, Muellner-Riehl AN, Michalak I, Tkach N, Hoffmann MH, Röser M, Sun H, Favre A (2017). In and out of the Qinghai-Tibet plateau: divergence time estimation and historical biogeography of the large arctic-alpine genus *Saxifraga* L. J Biogeogr.

[47] Gao Q-B, Li Y, Gengji Z-M, Gornall RJ, Wang J-L, Liu H-R, Jia L-K, Chen S-L (2017). Population genetic differentiation and taxonomic suggestion of three closely related species of *Saxifraga* (Saxifragaceae) from southern Tibet and the Hengduan Mountains. Frontiers Pl Sci.

[48] White TJ, Bruns T, Lee S, Taylor J, Innis MA, Gelfand DH, Sninsky JJ, White TJ (1990). Amplification and direct sequencing of fungal ribosomal RNA genes for phylogenetics. PCR protocols: a guide to methods and applications.

[49] Taberlet P, Gielly L, Pautou G, Bouvet J (1991). Universal primers for amplification of three non-coding regions of chloroplast DNA. Pl Molec Biol.

[50] Shaw J, Lickey EB, Schilling EE, Small RL (2007). Comparison of whole chloroplast genome sequences to choose noncoding regions for phylogenetic studies in angiosperms: the tortoise and the hare III. Amer J Bot..

[51] Larkin MA, Blackshields G, Brown NP, Chenna R, McGettigan PA, McWilliam H, Valentin F, Wallace IM, Wilm A, Lopez R, Thompson JD, Gibson TJ, Higgins DG (2007). ClustalW and ClustalX version 2.0. Bioinformatics.

[52] Kearse M, Moir R, Wilson A, Stones-Havas S, Cheung M, Sturrock S, Buxton S, Cooper A, Markowitz S, Duran C, Thierer T, Ashton B, Mentjies P, Drummond A (2012). Geneious basic: an integrated and extendable desktop software platform for the organization and analysis of sequence data. Bioinformatics.

[53] Suchan T, Talavera G, Sáez L, Ronikier M, Vila R. Pollen metabarcoding as a tool for tracking long-distance insect migrations. Mol Ecol Resour. 2018. 10.1101/312363. 10.1111/1755-0998.12948 30267472

[54] Chen S, Yao H, Han J (2010). Validation of the ITS2 region as a novel DNA barcode for identifying medicinal plant species. PLoS One.

[55] Fazekas AJ, Kuzmina ML, Newmaster SG, Hollingsworth PM, Kress W, Erickson D (2012). DNA barcoding methods for land plants. DNA barcodes. Methods in molecular biology (methods and protocols).

[56] Keller A, Danner N, Grimmer G, Ankenbrand M, von der Ohe K, von der Ohe W (2015). Evaluating multiplexed next-generation sequencing as a method in palynology for mixed pollen samples. Plant Biol.

[57] Sickel W, Ankenbrand MJ, Grimmer G, Holzschuh A, Härtel S, Lanzen J, Steffan-Dewenter I, Keller A (2015). Increased efficiency in identifying mixed pollen samples by meta-barcoding with a dual-indexing approach. BMC Ecol.

[58] Fierer N, Hamady M, Lauber CL, Knight R (2008). The influence of sex, handedness, and washing on the diversity of hand surface bacteria. Proc Natl Acad Sci U S A.

[59] Zhang J, Kobert K, Flouri T, Stamatakis A (2014). PEAR: a fast and accurate Illumina paired-end reAd mergeR. Bioinformatics.

[60] Martin M (2011). Cutadapt removes adapter sequences from high-throughput sequencing reads. EMBnet J.

[61] Rognes T, Flouri T, Nichols B, Quince C, Mahé F (2016). VSEARCH: a versatile open source tool for metagenomics. PeerJ.

[62] Stamatakis A (2014). RAxML version 8: a tool for phylogenetic analysis and post-analysis of large phylogenies. Bioinformatics.

[63] Miller MA, Pfeiffer W, Schwartz T. Creating the CIPRES Science Gateway for inference of large phylogenetic trees. In: Proceedings of the gateway computing environments workshop (GCE), New Orleans, Louisiana, 14 Nov. 2010. Piscataway: IEEE; 2010. p. 45–52.

[64] Huelsenbeck JP, Ronquist F. MrBayes: Bayesian inference of phylogeny. Ver. 3.1.2. 2006. http://mrbayes.csit.fsu.edu. Accessed 20 Mar 2018.

[65] Swofford DL (2002). PAUP*. Phylogenetic analysis using parsimony (*and other methods), version 4.

[66] Drummond AJ, Suchard MA, Xie D, Rambaut A (2012). Bayesian phylogenetics with BEAUti and the BEAST 1.7. Molec Biol Evol.

[67] Rambaut A, Suchard MA, Xie D, Drummond AJ. Tracer v1.6. 2014. http://tree.bio.ed.ac.uk/software/tracer/. Accessed 26 Jun 2018.

[68] Brochmann C, Nilsson T, Gabrielsen TM (1996). A classic example of postglacial allopolyploid speciation re-examined using RAPD markers and nucleotide sequences: *Saxifraga osloensis* (Saxifragaceae). Symb Bot Upsal.

[69] Rice A, Glick L, Abadi S, Einhorn M, Kopelman NM, Salman-Minkov A, Mayzel J, Chay O, Mayrose I (2015). The chromosome counts database (CCDB) – a community resource of plant chromosome numbers. New Phytol.

[70] Zhmylev PY (2004). Genus *Saxifraga* L. (Saxifragaceae): biomorphology, systematics and evolution of the life forms.

[71] Pawłowska S (1953). O kilku skalnicach karpackich i bałkańskich. De nonnulIis Saxifragis carpaticis et balcanicis. Acta Soc Bot Pol.

[72] Gornall RJ (1986). Trichome anatomy and taxonomy of *Saxifraga* (Saxifragaceae). Nordic J Bot.

[73] Engler HGA (1872). Monographie der Gattung *Saxifraga* L, mit besonderer Berücksichtigung der geographischen Verhältnisse.

[74] Engler HGA, Irmscher E. Saxifragaceae - *Saxifraga* I. In: Engler HGA, editor. Pflanzenreich, vol. 67 (IV, 117), Saxifragaceae - *Saxifraga*. Leipzig: Engelmann; 1916. p. 1–448.

[75] Ball J (1846). Adnotatio in speciem novam generis *Saxifraga*. Bot Zeitung (Berlin).

[76] Winterfeld G, Schneider J, Röser M (2009). Allopolyploid origin of Mediterranean species in *Helictotrichon* (Poaceae) and its consequences for karyotype repatterning and homogenisation of rDNA repeat units. Syst Biodivers.

[77] Kotseruba V, Pistrick K, Blattner FR, Kumke K, Weiss O, Rutten T, Fuchs J, Endo T, Nasuda S, Ghukasyan A, Houben A (2010). The evolution of the hexaploid grass *Zingeria kochii* (Mez) Tzvel. (2*n* = 12) was accompanied by complex hybridization and uniparental loss of ribosomal DNA. Mol Phylogenet Evol.

[78] Nieto Feliner G, Rosselló JA, Wendel JF (2012). Concerted evolution of multigene families and homeologous recombination. Plant genome diversity.

[79] Winterfeld G, Schneider J, Perner K, Röser M (2012). Origin of highly polyploids: different pathways of auto- and allopolyploidy in 12–18*x* species of *Avenula* (Poaceae). Int J Pl Sci..

[80] Weiss-Schneeweiss H, Emadzade K, Jang TS, Schneeweiss GM (2013). Evolutionary consequences, constraints and potential of polyploidy in plants. Cytogenet Genome Res.

[81] Wölk A, Winterfeld G, Röser M (2015). Genome evolution in a Mediterranean species complex: phylogeny and cytogenetics of *Helictotrichon* (Poaceae) allopolyploids based on nuclear DNA sequences (rDNA, topoisomerase gene) and FISH. Syst Biodivers.

[82] Brochmann C, Xiang Q-Y, Brunsfeld SJ, Soltis DE, Soltis PS (1998). Molecular evidence for polyploid origins in *Saxifraga* (Saxifragaceae): the narrow arctic endemic *S. svalbardensis* and its widespread allies. Amer J Bot..

[83] Marks L, Dzierżek J, Janiszewski R, Kaczorowski J, Lindner L, Majecka A, Makos M, Szymanek M, Tołoczko-Pasek A, Woronko B (2016). Quaternary stratigraphy and palaeogeography of Poland. Acta Geol Pol.

[84] Zając A, Zając M, editors. Atlas rozmieszczenia roślin naczyniowych w Polsce. Distribution atlas of vascular plants in Poland. 2001. https://atlas-roslin.pl/. Accessed 2 Nov 2017.

[85] Jasičová M, Futák J, Bertová L (1985). *Saxifraga* L. Flóra slovenska.

[86] Folk RA, Soltis PS, Soltis DE, Guralnick R (2018). New prospects in the detection and comparative analysis of hybridization in the tree of life. Amer J Bot.

[87] Mirek Z, Piękoś-Mirkowa H (1992). Plant cover of the polish Tatra Mountains (S Poland). Veröff Geobot Inst ETH, Stiftung Rübel, Zürich.

[88] Mai DH (1995). Tertiäre Vegetationsgeschichte Europas. Methoden und Ergebnisse.

[89] Milne RI, Abbott RJ (2002). The origin and evolution of tertiary relict floras. Advances Bot Res.

[90] Lendvay B, Kadereit JW, Westberg E, Cornejo C, Pedryc A, Höhn M (2016). Phylogeography of *Syringa josikaea* (Oleaceae): Early Pleistocene divergence from east Asian relatives and survival in small populations in the Carpathians. Biol J Linn Soc.

[91] Puşcaş M, Choler P, Tribsch A, Gielly L, Rioux D, Gaudeul M, Taberlet P (2008). Post-glacial history of the dominant alpine sedge *Carex curvula* in the European alpine system inferred from nuclear and chloroplast markers. Molec Ecol..

[92] Šrámková-Fuxová G, Záveská E, Kolář F, Lučanová M, Španiel S, Marhold K (2017). Range-wide genetic structure of *Arabidopsis halleri* (Brassicaceae): glacial persistence in multiple refugia and origin of the northern hemisphere disjunction. Bot J Linn Soc.

[93] Pfiffner OA (2015). Geologie der Alpen.

[94] Tribsch A, Schönswetter P (2003). Patterns of endemism and comparative phylogeography confirm palaeoenvironmental evidence for Pleistocene refugia in the eastern Alps. Taxon.

[95] Tribsch A, Schönswetter P, Stuessy TF (2002). *Saponaria pumila* (Caryophyllaceae) and the ice age in the European Alps. Amer J Bot..

[96] Schönswetter P, Tribsch A, Schneeweiss GM, Niklfeld H (2003). Disjunction in relict alpine plants: phylogeography of *Androsace brevis* and *A. wulfeniana* (Primulaceae). Bot J Linn Soc.

[97] Thiers B. Index Herbariorum: a global directory of public herbaria and associated staff. New York botanical Garden’s virtual herbarium. Continuously updated. http://sweetgum.nybg.org/science/ih/.

