# Supplementary material for: Contrasting evolutionary origins of two mountain endemics: Saxifraga wahlenbergii (Western Carpathians) and S. styriaca (Eastern Alps)
Source: BMC Evol Biol. 2019 Jan 11;19:18. doi: 10.1186/s12862-019-1355-x (PMC6329101; doi:10.1186/s12862-019-1355-x)
Supplement: Supplementary file 1 — Table S1. Taxa included in the phylogenetic analysis. ENA/GenBank accession numbers are given for all ITS, trnL–trnF and rpl32–trnL Sanger sequences used in this study, followed by available ITS2 sequences obtained from NGS (without ENA/GenBank entry). They are denoted as “ITS2 variants A–D”. Missing sequence data are indicated by “NA” (not available). Voucher data (country and region, collector and collection number, herbarium code according to Thiers et al. [97]) are provided for newly generated sequences, which are in bold print. MR: herbarium of G. & S. Miehe deposited at the Institute of Geography, University Marburg, Germany. (PDF 502 kb) [file 12862_2019_1355_MOESM1_ESM.pdf]

Additional file 1

Contrasting evolutionary origins of two mountain endemics: *Saxifraga wahlenbergii* (Western Carpathians) and *S. styriaca* (Eastern Alps)

Natalia Tkach[1*¶], Martin Röser[1¶], Tomasz Suchan[2], Elżbieta Cieślak[2], Peter Schönswetter[3], Michał Ronikier[2*]

[1] Martin Luther University Halle-Wittenberg, Institute of Biology, Geobotany and Botanical Garden, Neuwerk 21, 06108 Halle, Germany

[2] W. Szafer Institute of Botany, Polish Academy of Sciences, Lubicz 46, 31-512, Krakow, Poland

[3] University of Innsbruck, Department of Botany, Sternwartestraße 15, 6020 Innsbruck, Austria

* Corresponding authors: m.ronikier@botany.pl, natalia.tkach@botanik.uni-halle.de

¶ These authors contributed equally to this work.

Table S1. Taxa included in the phylogenetic analysis. ENA/GenBank accession numbers are given for all ITS, *trn*L–*trn*F and *rpl*32–*trn*L Sanger sequences used in this study, followed by available ITS2 sequences obtained from NGS (without ENA/GenBank entry). They are denoted as "ITS2 variants A–D". Missing sequence data are indicated by "NA" (not available). Voucher data (country and region, collector and collection number, herbarium code according to Thiers et al. [91]) are provided for newly generated sequences, which are in bold print. MR: herbarium of G. & S. Miehe deposited at the Institute of Geography, University Marburg, Germany.

Taxon name	ITS	ITS2 variants	trnL-trnF	rpl32	Provenance, voucher information of the new sequenced samples
**Grossulariaceae**					
Ribes amarum McClatchie	KU524089		KU524330	NA	
R. horridum Rupr. ex Maxim.	KU524090		KU524333	NA	
**Iteaceae**					
Choristylis rhamnoides Harv.	KU524085		KU524323	NA	
Itea virginica L.	AY231368		AF374818	NA	
I. yunnanensis Franch.	JN102242		JN102291	NA	
Pterostemon rotundifolius Ramírez	AY231369		AF374817	NA	
**Penthoraceae**					
Penthorum chinense Pursch	KC988286		NC_023086	NC_023086	
**Saxifragaceae**					
Astilbe microphylla Knoll	FJ653634, FJ653651		AF374814	NA	
Astilboides tabularis (Hemsl.) Engl.	JQ895221		AF374793	NA	
Bensoniella oregona (Abrams & Bacig.) C.V.Morton	AF158953		NA	KT755705	
Cascadia nuttallii (Small) A.M.Johnson (1)	LM654340		LM654424	**LT971015**	
C. nuttallii (Small) A.M.Johnson (2)	LM654339		LM654423	**LT971016**	
Chrysosplenium iowense Rydb.	Alignment (Xiang et al. 2012[1])		AF374790	NA	
Conimitella williamsii (D.C.Eaton) Rydb.	AB292020		NA	KT755694	
Darmera peltata (Torr. ex Benth.) Voss	AB292041		AF374795	KT755657	
Elmera racemosa (S.Watson) Rydb.	AB248849		AF374804	KT755658	
Heuchera micrantha Douglas ex Lindl.	AB248853		AF374806	KT755702	
Jepsonia parryi (Torr.) Small	U51262		AF374808	NA	

Leptarrhena pyrolifolia (D.Don) R.Br. ex Ser.	Alignment (Xiang et al. 2012)		AF374812	NA	
Lithophragma trifoliatum Eastw.	AF158951		NA	NA	
Micranthes brachypetala (Malyschev) Gornall & H.Ohba	LM654344		LM654428	**LT971017**	
Micranthes californica (Greene) Small	LM654347		LM654431	**LT971018**	
M. calycina (Sternb.) Gornall & H.Ohba	LM654349		LM654433	**LT971019**	
M. divaricata (Engl. & Irmsch.) Losinsk.	LM654354		LM654438	**LT971020**	
M. ferruginea (Graham) Brouillet & Gornall	LM654357		LM654441	**LT971021**	
M. integrifolia (Hook.) Small	LM654365		LM654449	**LT971022**	
M. lyallii (Engl.) Small subsp. *lyallii*	LM654368		LM654452	**LT971023**	
M. melanocentra (Franch.) Losinsk.	LM654372		LM654456	**LT971024**	
M. merkii (Fisch. ex Sternb.) Elven & D.F.Murray subsp. *merkii*	LM654374		LM654457	**LT971025**	
M. micranthidifolia (Haw.) Small	LM654375		LM654459	**LT971026**	
M. nelsoniana (D.Don) Small	LM654379		LM654463	**LT971027**	
M. nivalis (L.) Small	LM654384		LM654468	**LT971028**	
M. nudicaulis (D.Don) Gornall & H.Ohba	LM654385		LM654469	**LT971029**	
M. pensylvanica (L.) Haw.	LM654391		LM654475	**LT971030**	
M. razshivinii (Zhmylev) Brouillet & Gornall	LM654398		LM654482	**LT971031**	
M. redofskyi (Adams) Elven & D.F.Murray	LM654399		LM654483	**LT971032**	
M. reflexa (Hook.) Small	LM654401		LM654485	**LT971033**	
M. stellaris (L.) Galasso, Banfi & Soldano	LM654407		LM654491	**LT971034**	
M. tilingiana (Regel & Til.) Kom.	LM654412		LM654496	**LT971035**	
M. tolmiei (Torr. & A.Gray) Brouillet & Gornall	LM654413		LM654497	**LT971036**	
Mitella nuda L.	AB163495		AB116715	NA	
Mukdenia rossii (Oliv.) Koidz.	Alignment (Xiang et al. 2012)		AF374794	NA	
Oresitrophe rupifraga Bunge	JN102231		JN102280	NA	
Peltoboykinia tellimoides (Maxim.) H.Hara	AB248847		AF374789	KT755740	
Rodgersia pinnata Franch.	U51264		AF374791	KT755741	
Saniculiphyllum guangxiense C.Y.Wu & T.C.Ku	JN102233		JN102282	NA	
Saxifraga adscendens L. (1)	LN812341	**A**	LN812575	**LT971037**	Romania, Jud. Hunedoara; M. Röser 9710 (HAL)

S. adscendens L. (2)	**LT970992**	**A**	**LT970892**	**LT971038**	Romania, Jud. Hunedoara; M. Röser 9825 (HAL)
S. afghanica L.	LN812342		LN812576	NA	
S. aizoides L.	LN812343		LN812577	**LT971039**	Russia, cult. Polar-Alpine Botanical Garden-Institute (PABGI): Kirovsk; N. Tkach (HAL)
S. alberti Regel & Schmalh.	LN812344		LN812578	NA	
S. aleutica Hultén	LN812345		LN812579	**LT971040**	USA, Alaska; S. Talbot & S.L. Talbot 489 (NY)
S. alpigena Harry Sm.	LN812346		LN812580	**LT971041**	Germany, cult. Brockengarten Harz (11-B-0080); N. Tkach 225 & M. Röser (HAL)
S. anadyrensis Losinsk. (1)	LN812347		LN812581	NA	
S. anadyrensis Losinsk. (2)	LN812348		LN812582	**LT971042**	Russia, Chukotka; A.A. Korobkov & N.A. Sekretareva (LE)
S. andersonii Engl.	LN812349		LN812583	**LT971043**	Germany, cult. Brockengarten Harz (09-B-0011); N. Tkach 228 & M. Röser (HAL)
S. androsacea L. (1)	**LT970993**	**A**	NA	**LT971044**	Russia, Sayan Mountains; I. Kasnoborov & V. Osadczy 4980 (LE)
S. androsacea L. (2)	NA	**A, B**	NA	NA	Slovakia, Carpathians, Western Tatras; M. Ronikier & A. Ronikier (KRAM)
S. androsacea L. (3)	NA	**A, B, C, D**	NA	NA	Spain, Lleida, Aiguestortes; B. Frajman, P. Schönswetter (W)
S. angustata Harry Sm.	LN812351		LN812585	**LT971045**	China, NW Sichuan; G. & S. Miehe & U. Wuendisch 94-404-14 (Germany: Marburg)
S. aphylla Sternb. (1)	LN812352		LN812586	**LT971046**	Switzerland, Canton of Grisons; M. Röser 7045 (HAL)
S. aphylla Sternb. (2)	**LT970994**	**A**	**LT970893**	**LT971047**	Germany, Eastern Alps, Wetterstein mountains; H. Meusel (HAL0075075)
S. aquatica Lapeyr.	AF261161		KU524334	NA	
S. arachnoidea Sternb.	LN812353		LN812587	**LT971048**	Italy, Prov. Brescia; M. Röser 10259 (HAL)
S. aretioides Lapeyr.	LN812354		LN812588	**LT971049**	Germany, cult. Arctic-Alpine Garden Chemnitz (s.n.); N. Tkach 209 & M. Röser (HAL)
S. aristulata Hook.f. & Thomson	LN812355		LN812589	**LT971050**	China, Xizang; G. & S. Miehe 99-134-20 (Germany: Marburg)
S. aspera L.	LN812356		LN812590	**LT971051**	Austria, Vorarlberg; M. Röser 5839 (HAL)
S. aurantiaca Franch.	LN812357		LN812591	**LT971052**	China, Xizang; G. & S. Miehe & U. Wuendisch 94-339-12c (Germany: Marburg)
S. babiana T.E.Díaz & Fern.Prieto	LN812358		LN812592	**LT971053**	Germany, cult. Arctic-Alpine Garden Chemnitz (s.n.); N. Tkach 210 & M. Röser (HAL)
S. bergenioides C.Marquand	LN812359		LN812593	**LT971054**	Bhutan, Gasa; G. & S. Miehe 00-357-03 (Germany: Marburg)
S. berica (Bég.) D.A.Webb	LN812360		LN812594	**LT971055**	Italy, Monti Berici; H. Meltesics (LE)
S. bicuspidata Hook.f.	AF374819, AF374820		AF374776	NA	
S. biflora All.	LN812361		LN812595	NA	
S. biternata Boiss.	LN812362		LN812596	**LT971056**	Germany, cult. Botanical Garden Muenchen-Nymphenburg (ES-0-M-2006/1581); N. Tkach 260 & M. Röser (HAL)
S. blepharophylla A.Kern. ex Hayek	AF504545		NA	NA	

S. bourgaeana Boiss. & Reut.	LN812364	LN812598	**LT971057**	Spain, Malaga; M. Röser 4682 (HAL)
S. brachypoda D.Don	LN812365	LN812599	**LT971058**	Bhutan, Gasa; G. & S. Miehe 00-385-10 (Germany: Marburg)
S. bracteata D.Don (1)	LN812366	NA	NA	
S. bracteata D.Don (2)	LN812367	LN812601	**LT971059**	USA, Alaska; T. Kelso, J. Flock & M. Colson 235 (NY)
S. bronchialis L. subsp. *bronchialis*	LN812369	LN812603	NA	
S. bronchialis L. subsp. *austromontana* (Wiegand) Piper	LN812368	LN812602	**LT971060**	Germany, cult. Botanical Garden Muenchen-Nymphenburg (US-0-M-2006/0173); N. Tkach 259 & M. Röser (HAL)
S. brunonis Wall. ex Ser. (1)	JN102236	NA	NA	
S. brunonis Wall. ex Ser. (2)	LN812370	LN812604	**LT971061**	Bhutan, Gasa; G. & S. Miehe 00-293-01 (Germany: Marburg)
S. bryoides L.	LN812371	LN812605	**LT971062**	Romania, Jud. Hunedoara; M. Röser 9602 (HAL)
S. bulbifera L.	LN812372	LN812606	**LT971063**	Austria, Lower Austria; M. Röser 9124 (HAL)
S. caesia L.	LN812373	LN812607	**LT971064**	Italy, Prov. Brescia; M. Röser 10264 (HAL)
S. callosa Sm. ex Dicks.	LN812374	LN812608	NA	
S. callosa Sm. ex Dicks. subsp. *catalaunica* (Boiss. & Reut.) D.A.Webb	LN812380	LN812614	**LT971074**	Germany, cult. Arctic-Alpine Garden Chemnitz (AC1547); N. Tkach 212 & M. Röser (HAL)
S. camposii Boiss. & Reut. var. *leptophylla* Willk.	LN812375	LN812609	**LT971065**	Spain, Pena Lucas. Almeria; A. Segura Zubizarreta (LE)
S. canaliculata Boiss. & Reut. ex Engl.	LN812376	LN812610	**LT971066**	Germany, cult. Arctic-Alpine Garden Chemnitz (AC-1313); N. Tkach 211 & M. Röser (HAL)
S. carpatica Rchb.	LN812377	LN812611	**LT971067**	Romania, Jud. Hunedoara; M. Röser 9676 (HAL)
S. carpetana Boiss. & Reut.	AF261168	NA	NA	
S. cartilaginea Willd.	LN812378	LN812612	NA	
S. caspica Sipliv.	LN812379	LN812613	**LT971068**	Russia, E Caucasus; V. Prima, A. Syunyakov (LE)
S. caucasica Sommier & Levier	LN812381	LN812615	**LT971069**	Russia, Caucasus; G.L. Kudryashova (LE)
S. caulescens Sipliv.	KF196325	KF196375	NA	
S. caveana W.W.Sm.	LN812382	LN812616	**LT971070**	Bhutan, Gasa; G. & S. Miehe 00-303-12 (Germany: Marburg)
S. cebennensis Rouy & E.G.Camus	LN812383	LN812617	**LT971071**	Germany, cult. Botanical Garden Muenchen-Nymphenburg (FR-0-M-2003/1261); N. Tkach 261 & M. Röser (HALl)
S. cernua L. (1)	EU158861	AF374779	NA	
S. cernua L. (2)	LN812384	LN812618	NA	
S. cespitosa L. subsp. *cespitosa*	LN812385	LN812619	**LT971072**	Russia, Taymyr; Yu.P. Kozhevnikov (LE)
S. cespitosa L. subsp. *monticola* (Small) A.E.Porsild	LN812386	LN812620	**LT971073**	Russia, Wrangel Island; V.V. Petrovsky & P.G. Zhukova (LE)
S. charadzeae Otsch.	LN812387	LN812621	**LT971076**	Russia, Caucasus; T.N. Popova & G.L. Kudryashova (LE)
S. cherlerioides D.Don var. *cherlerioides*	LN812389	LN812623	NA	
S. cherlerioides D.Don var. *rebunshirensis* Hara	LN812388	LN812622	**LT971077**	Germany, cult. Botanical Garden Tuebingen (AC 691); N. Tkach 252 & M. Röser (HAL)
S. chrysantha A.Gray	LN812390	LN812624	**LT971078**	USA, Colorado; G.N. Jones (LE)

S. cintrana Kuzinsky ex Willk.	AF261171		NA	NA	
S. cochlearis Rchb.	LN812391		LN812625	**LT971079**	Germany, cult. Arctic-Alpine Garden Chemnitz (AC-1372); N. Tkach 213 & M. Röser (HAL)
S. colchica Albov	LN812392		LN812626	NA	
S. columnaris Schmalh.	LN812393		LN812627	**LT971080**	Russia, Caucasus; S. Shkhagansoev (LE)
S. conifera Coss. & Durieu (1)	AJ233865		NA	NA	
S. conifera Coss. & Durieu (2)	LN812394		LN812628	NA	
S. consanguinea W.W.Sm.	LN812395		LN812629	**LT971081**	China, Xizang; G. & S. Miehe & U. Wuendisch 94-344-12 (Germany: Marburg)
S. continentalis D.A.Webb	LN812396		LN812630	**LT971082**	Spain, Moncayo; A. Segura Zubizarreta (LE)
S. contraria Harry Sm. f. *rubella* Harry Sm.	LN812397		LN812631	**LT971083**	Bhutan, Gasa; G. & S. Miehe 00-322-02 (Germany: Marburg)
S. coriifolia Grossh.	LN812398		LN812632	**LT971084**	Georgia, Abkhazia; E. Mordakh (LE)
S. corsica Gren. & Godron	LN812399		LN812633	**LT971085**	Italy, Sardinia; M. Röser 10171 (HAL)
S. cortusifolia Siebold & Zucc.	NA		LN812634	**LT971086**	Russia, Far East: Ostrov Falshivyy; E. Pobedimova & G. Konovalova (LE)
S. corymbosa Luce	LN812400		LN812635	**LT971087**	Romania, Jud. Hunedoara; M. Röser 9855 (HAL)
S. cossoniana (Boiss. & Reut.) D.A.Webb	AF261172		NA	NA	
S. cotyledon L.	LN812401		LN812636	**LT971088**	Spain, Prov. Huesca; M. Röser 10572 (HAL)
S. crustata Vest.	LN812402		LN812637	**LT971089**	Germany, cult. Botanical Garden MLU Halle-Wittenberg; N. Tkach 79 (HAL)
S. cuneata Willd.	LN812403		LN812638	**LT971090**	Germany, cult. Brockengarten Harz (11-B-0134); N. Tkach 230 & M. Röser (HAL)
S. cuneifolia L.	LN812404		LN812639	**LT971091**	Germany, cult. Botanical Garden MLU Halle-Wittenberg; N. Tkach 71 (HAL)
S. cymbalaria L.	LN812405		LN812640	**LT971092**	Russia, Krasnodarskiy kray; T.N. Popova & S.V. Bondarenko (LE)
S. depressa Sternb. (1)	AF261173		NA	NA	
S. depressa Sternb. (2)	NA	**A, B**	NA	NA	Italy, Belluno, Dolomites; G. Schneeweiss & P. Schönswetter 5247 (WU)
S. derbekii Sipliv.	LN812406		LN812641	**LT971093**	Russia, Magadanskaya obl.; A.T. Reutt (LE)
S. desoulavyi Oett.	LN812407		LN812642	**LT971094**	Germany, cult. Arctic-Alpine Garden Chemnitz (AC-1353); N. Tkach 214 & M. Röser (HAL)
S. diapensioides Bell.	LN812408		LN812643	**LT971095**	Germany, cult. Brockengarten Harz (2000-B-08); N. Tkach 231 & M. Röser (HAL)
S. dichotoma Willd. ex Sternb.	LN812409		LN812644	**LT971096**	Spain, Sierra de Pela; Rivas Goday (HAL0051497)
S. dingqingensis J.T.Pan	EU158857		NA	NA	
S. dinnikii Schmalh. ex Akinf.	LN812410		LN812645	NA	
S. discolor Velen.	LN812411	**A**	NA	**LT971097**	Bulgaria, Pirin; W. Hilbig (HAL0065463)
S. diversifolia Wall. ex Ser. var. *diversifolia*	LN812412		LN812646	**LT971098**	China, Sichuan; G. & S. Miehe & U. Wuendisch 94-510-29 (Germany: Marburg)

S. duthiei Gand.	LN812413	LN812647	**LT971099**	Germany, cult. Brockengarten Harz (06-B-0111); N. Tkach 223 & M. Röser (HAL)
S. egregia Engl.	EU158836	NA	NA	
S. engleriana Harry Sm.	LN812414	LN812648	**LT971100**	China, Xizang; G. & S. Miehe 9541/03 (Germany: Marburg)
S. erioblasta Boiss. & Reut.	LN812415	LN812649	**LT971101**	Spain, Granada; M. Röser 3251 (HAL)
S. eschscholtzii Sternb. (1)	LN812416	LN812650	**LT971102**	Russia, Chukotka; P. Zhmylev, E.Yu. Norkina & T.V. Plieva (LE)
S. eschscholtzii Sternb. (2)	NA	LN812651	**LT971103**	USA, Alaska; M.H. Hoffmann 11/55 (HAL)
S. exarata Vill.	LN812417	LN812652	**LT971104**	Italy, Piemont; M. Röser 6547 (HAL)
S. facchinii W.D.J. Koch	**LT970995**	**LT970894**	**LT971105**	Italy, Belluno, Dolomites; C. Argenti (FIPF)
S. federici-augusti Biasoletti subsp. *grisebachii* (Degen & Dörfl.) D.A.Webb	LN812443	LN812678	**LT971106**	Germany, cult. Arctic-Alpine Garden Chemnitz (WR9905); N. Tkach 216 & M. Röser (HAL)
S. felineri P.Vargas	LN812419	LN812654	**LT971107**	Germany, cult. Arctic-Alpine Garden Chemnitz (AC-1317); N. Tkach 207 & M. Röser (HAL)
S. ferdinandi-coburgi Kellerer & Sund.	LN812420	LN812655	**LT971108**	Germany, cult. Brockengarten Harz (05-B-0111); N. Tkach 232 & M. Röser (HAL)
S. filicaulis Wall. ex Ser.	LN812421	LN812656	**LT971109**	China, Xizang; Miehe 97-064-10, Huang, Otsu & Tunsu (Germany: Marburg)
S. finitima W.W.Sm.	LN812422	LN812657	**LT971110**	China, Xizang; G. Miehe & U. Wuendisch 94-118-03 (Germany: Marburg)
S. firma Litv. ex Losinsk. (1)	LN812423	NA	NA	
S. firma Litv. ex Losinsk. (2)	LN812424	LN812659	**LT971111**	Russia, Yakutia; Yu.P. Kozhevnikov & M.D. Andreeva (498) (LE)
S. flagellaris Willd. subsp. *crandallii* (Gand.) Hultén	LN812425	LN812660	**LT971112**	USA, Colorado; R.E. Brooks 20451 (NY)
S. flagellaris Willd. subsp. *crassiflagellata* Hultén	LN812426	LN812661	**LT971113**	Pakistan, Shinghai Gah to Pahot Gali; G. & S. Miehe 1008 (Germany: Marburg)
S. flagellaris Willd. subsp. *stenophylla* (Royle) Hultén	LN812427	NA	**LT971114**	Pakistan, Hunza Valley; G. & S. Miehe 6420 (Germany: Marburg)
S. flexuosa Sternb.	LN812516	LN812751	**LT971115**	Russia, Kamchatka; E. Hulten (LE)
S. florulenta Moretti	AF087621, AF087591	NA	NA	
S. fortunei Hook. var. *fortunei*	NA	LN812663	**LT971116**	Russia, Kunashir; E. Egorova & L. Kolchanova (LE)
S. fortunei Hook. var. *incisolobata* (Engl. & Irmsch.) Nakai.	LN812428	LN812662	**LT971117**	Germany, cult. Arctic-Alpine Garden Chemnitz (AC-1422); N. Tkach 215 & M. Röser (HAL)
S. fragilis Schrank (1)	LN812429	LN812664	NA	
S. fragilis Schrank (2)	AF261176	NA	NA	
S. fragosoi Sennen	LN812430	LN812665	**LT971118**	Germany, cult. Arctic-Alpine Garden Chemnitz (AC-1847); N. Tkach 206 & M. Röser (HAL)
S. funstonii (Small) Fedde (1)	LN812431	LN812666	**LT971119**	Russia, Sakhalin; A. Taran (LE)
S. funstonii (Small) Fedde (2)	LN812432	LN812667	**LT971120**	Russia, Chukotka; M.P. Andreev, E.Yu. Norkina & V.V. Petrovsky

				(LE)
S. gemmigera Engl. var. *gemmuligera* (Engl.) & Gornall	LN812433	LN812668	**LT971121**	China, Sichuan; G. & S. Miehe & U. Wuendisch 94-390-09 (Germany: Marburg)
S. gemmipara Franch.	EU158859	NA	NA	
S. gemmulosa Boiss.	LN812434	LN812669	**LT971122**	Spain, Malaga; M. Röser 4738 (HAL)
S. genesiana P.Vargas	AF261178	NA	NA	
S. georgei J.Anthony	LN812435	LN812670	**LT971123**	Germany, cult. Brockengarten Harz (10-B-0080); N. Tkach 233 & M. Röser (HAL)
S. geranioides L.	LN812436	LN812671	**LT971124**	France, Pyrenees-Orient; M. Röser 10531 (HAL)
S. globulifera Desf. (1)	LN812437	LN812672	**LT971125**	Spain, Cadiz; M. Röser 4622 (HAL)
S. globulifera Desf. (2)	LN812438	LN812673	**LT971126**	Algeria, Chellala; V.P. Botschantzev (LE)
S. gouldii C.E.C.Fisch.	LN812439	LN812674	**LT971127**	Bhutan, Paro; G. & S. Miehe 00-160-35 (Germany: Marburg)
S. graeca Boiss.	LN812440	LN812675	NA	
S. granulata L.	LN812441	LN812676	**LT971128**	Austria, Lower Austria; M. Röser 7437 (HAL)
S. granulifera Harry Sm.	LN812442	LN812677	**LT971129**	Bhutan, Paro; G. & S. Miehe 00-160-14 (Germany: Marburg)
S. haenseleri Boiss. & Reut.	AF261180	NA	NA	
S. hariotii Luizet & Soulie	AF261181	NA	NA	
S. hederacea L. (1)	AF261182	NA	NA	
S. hederacea L. (2)	LN812444	LN812679	NA	
S. heleonastes Harry Sm.	LN812445	LN812680	**LT971130**	China, Xizang; G. & S. Miehe 03-094-26 (Germany: Marburg)
S. heterotricha C.Marquand & Airy Shaw	LN812446	LN812681	**LT971131**	China, Xizang; G. Miehe & U. Wuendisch 94-150-20 (Germany: Marburg)
S. hirculoides Decne. (2)	LN812574	LN812725	**LT971075**	Nepal, Dolpo; G. & S. Miehe 99-046-09 (Germany: Marburg)
S. hirculoides Decne. (1)	LN812447	LN812682	**LT971132**	Pakistan, Upper Kaghan Valley; G. & S. Miehe 570 (Germany: Marburg)
S. hirculus L. var. *hirculus* (1)	LN812449	LN812684	NA	
S. hirculus L. var. *hirculus* (2)	LN812450	LN812685	**LT971133**	Russia, Altai; M.H. Hoffmann (M36) (HAL)
S. hirculus L. var. *alpina* Engl.	LN812448	LN812683	**LT971134**	China, Xinjiang; G. & S. Miehe 5780 (Germany: Marburg)
S. hirsuta L.	LM654415	LM654499	NA	
S. hispidula D.Don (1)	JN102237	NA	NA	
S. hispidula D.Don (2)	LN812451	LN812686	**LT971135**	Bhutan, Thimphu; G. & S. Miehe 00-205-18 (Germany: Marburg)
S. hookeri Engl. & Irmsch.	EU158840	NA	NA	
S. hostii Tausch	LN812452	LN812687	**LT971136**	Germany, cult. Botanical Garden MLU Halle-Wittenberg; N. Tkach 70 (HAL)
S. hyperborea R.Br.	LN812453	LN812688	**LT971137**	Russia, Chukotka; A.A. Korobkov (LE)
S. hypnoides L. (1)	LN812454	LN812689	**LT971138**	Germany, cult. Botanical Garden MLU Halle-Wittenberg; N. Tkach 65 (HAL)
S. hypnoides L. (2)	**LT970996**	**LT970895**	**LT971139**	France, Pyrenees-Orient; M. Röser 10494 (HAL)

S. *implicans* Harry Sm.	LN812455		LN812690	**LT971140**	China, Xizang; G. & S. Miehe 10-27-02 (Germany: Marburg)
S. *insolens* Irmsch.	EU158841		NA	NA	
S. *intricata* Lapeyr.	AJ133030		NA	KJ774295	
S. *iranica* Bornm.	LN812456		LN812691	NA	
S. *irrigua* M.Bieb.	LN812457		LN812692	NA	
S. *isophylla* Harry Sm.	LN812458		LN812693	**LT971141**	China, Xizang; G. Miehe & U. Wuendisch 94-182-12 (Germany: Marburg)
S. *italica* D.A.Webb	LN812459	**A**	LN812694	NA	Italy, Gran Sasso; s. coll. (NEU346061)
S. *jacquemontiana* Decne.	LN812460		LN812695	**LT971142**	Bhutan, Gasa; G. & S. Miehe 00-367-09 (Germany: Marburg)
S. *juniperifolia* Adams	LM654416		LM654500	**LT971143**	
S. *kingdonii* C.Marquand	LN812461		LN812696	**LT971144**	China, Xizang; G. Miehe & U. Wuendisch 94-173-10 (Germany: Marburg)
S. *kingiana* Engl. & Irmsch.	EU158851		NA	NA	
S. *kolenatiana* Regel	LN812462		LN812697	**LT971145**	Georgia, Caucasus; Z. Klochkova (LE)
S. *komarovii* Losinsk.	LN812463		LN812698	**LT971146**	Tajikistan, Pamir; N.N. Tzvelev (LE)
S. *korshinskii* Kom.	LM654417		LM654501	**LT971147**	
S. *kotschyi* Boiss.	LN812464		LN812699	**LT971148**	Germany, cult. Brockengarten Harz (10-B-0014); N. Tkach 235 & M. Röser (HAL)
S. *kruhsiana* Fisch. ex Ser.	LN812465		LN812700	**LT971149**	Russia, Chukotka; A.A. Korobkov & N.A. Sekretareva (LE)
S. *lactea* Turcz.	LN812466		LN812701	**LT971150**	Russia, Yakutia; V.V. Petrovsky (LE)
S. *latepetiolata* Willk. (1)	LN812467		LN812702	NA	
S. *latepetiolata* Willk. (2)	AF261183		NA	NA	
S. *latiflora* Hook.f. & Thomson	LN812468		LN812703	**LT971151**	Bhutan, Wangdue-Phodrang; G. & S. Miehe 00-421-04 (Germany: Marburg)
S. *lilacina* Duthie	LN812469		LN812704	**LT971152**	Germany, cult. Botanical Garden MLU Halle-Wittenberg; N. Tkach 80 (HAL)
S. *lingulata* Bellardi	LN812470		LN812705	**LT971153**	Italy, Piemont; M. Röser 6664 (HAL)
S. *litangensis* Engl.	LN812471		LN812706	**LT971154**	China, Xizang; G. Miehe & U. Wuendisch 94-136-32 (Germany: Marburg)
S. *luteoviridis* Schott & Kotschy	LN812472		LN812707	**LT971155**	Germany, cult. Brockengarten Harz (11-B-0335); N. Tkach 236 & M. Röser (HAL)
S. *lychnitis* Hook.f. & Thomson	LN812473		LN812708	**LT971156**	Bhutan, Gasa; G. & S. Miehe 00-373-04 (Germany: Marburg)
S. *macrocalyx* Tolm.	LN812474		LN812709	**LT971157**	Russia, Altai; R.V. Kamelin et al. (LE)
S. *maderensis* D.Don	LN812475		NA	**LT971158**	Portugal, Madeira; K. Werner (HAL0079197)
S. *magellanica* Poir.	LN812476		LN812710	**LT971159**	Germany, cult. Botanical Garden Tuebingen (5539); N. Tkach 254 & M. Röser (HAL)
S. *marginata* Sternb. (1)	LN812478		LN812712	**LT971160**	Romania, Jud. Hunedoara; M. Röser 9695 (HAL)
S. *marginata* Sternb. (2)	LN812363		LN812597	**LT971161**	Germany, cult. Brockengarten Harz (98-B-2036); N. Tkach 229 & M.

					Röser (HAL)
S. marginata Sternb. var. *bubakii* (Rohlena) Horný	LN812477		LN812711	**LT971162**	Germany, cult. Arctic-Alpine Garden Chemnitz (s.n.); N. Tkach 217 & M. Röser (HAL)
S. matta-florida Harry Sm.	LN812479		LN812713	**LT971163**	Germany, cult. Brockengarten Harz (08-B-0111); N. Tkach 237 & M. Röser (HAL)
S. mertensiana Bong.	LM654418		LM654502	**LT971164**	
S. moncayensis D.A.Webb	LN812480		LN812714	**LT971165**	Germany, cult. Brockengarten Harz (02-B-0158); N. Tkach 238 & M. Röser (HAL)
S. moorcroftiana (Ser.) Wall. ex Sternb.	LN812482		LN812716	**LT971166**	Bhutan, Paro; G. & S. Miehe 00-177-02 (Germany: Marburg)
S. moschata Wulfen	LN812483		LN812717	**LT971167**	Germany, cult. Botanical Garden MLU Halle-Wittenberg; N. Tkach 82 (HAL)
S. mucronulata Royle	LN812484		LN812718	**LT971168**	China, Xizang; G. Miehe & U. Wuendisch 94-251-15 (Germany: Marburg)
S. mucronulatoides J.T.Pan	LN812485		LN812719	**LT971169**	Bhutan, Gasa; G. & S. Miehe 00-320-04 (Germany: Marburg)
S. mutata L.	LN812486		LN812720	**LT971170**	Italy, Lombardia; M. Röser 10412 (HAL)
S. nanella Engl. & Irmsch.	LN812487		LN812721	**LT971171**	China, Qinghai; G. & S. Miehe 9388/17 (Germany: Marburg)
S. nangxianensis J.T.Pan	EU158850		NA	NA	
S. nevadensis Boiss.	LN812488		LN812722	**LT971172**	Spain, Granada; M. Röser 3313 (HAL)
S. nigroglandulifera N.P.Balakr.	NA		LN812723	NA	
S. nipponica Makino	LN812489		LN812724	**LT971173**	Japan, Honshu; M. Togasi 1707 (LE)
S. nishidae Miyabe & Kudô	KF196324		KF196383	NA	
S. omolojensis A.P.Khokhr. (1)	LN812490		LN812726	**LT971174**	Russia, Magadanskaya obl.; A.T. Reutt (LE)
S. omolojensis A.P.Khokhr. (2)	LN812491		NA	NA	
S. oppositifolia L. (1)	AY354299		AF374782	NA	
S. oppositifolia L. (2)	LM654419		LM654503	**LT971175**	
S. oppositifolia L. subsp. *oppositifolia*	NA		LN812727	NA	
S. oppositifolia L. subsp. *asiatica* (Hayek) Engl. & Irmsch. (1)	LN812492		LN812728	NA	
S. oppositifolia L. subsp. *asiatica* (Hayek) Engl. & Irmsch. (2)	NA		LN812729	NA	
S. oppositifolia L. subsp. *smalliana* (Engl. & Irmsch.) Hultén	AF504544		NA	NA	
S. osloensis Knaben (1)	LN812493	**A**	LN812730	NA	Germany, cult. Botanical Garden Berlin; Schwerdtfeger 12431 (B100550421)
S. osloensis Knaben (2)	AF087638, AF087608		AF374788	NA	
S. paniculata Mill.	LN812494		LN812731	**LT971176**	Germany, cult. Botanical Garden MLU Halle-Wittenberg; N. Tkach 66 (HAL)
S. paradoxa Sternb.	LN812495		LN812732	**LT971177**	Austria, Carinthia; R. Freiherr von Benz 3095 (HAL0021485)

S. parnassifolia D.Don	LN812496		LN812733	**LT971178**	Bhutan, Thimphu; G. & S. Miehe 00-223-28 (Germany: Marburg)
S. pedemontana All. subsp. *cymosa* Engl.	LN812497		LN812734	**LT971179**	Romania, Jud. Hunedoara; M. Röser 9674 (HAL)
S. pentadactylis Lapeyr. (1)	LN812498		LN812735	**LT971180**	France, Pyrenees-Orient; M. Röser 10552 (HAL)
S. pentadactylis Lapeyr. (2)	LN812499		LN812736	**LT971181**	Spain, Pico de San Lorennzo; A. Segura Zubizarreta (LE)
S. pentadactylis Lapeyr. subsp. *willkommiana* Rivas Mart.	AY354308		NA	NA	
S. peplidifolia Franch.	EU158843		NA	NA	
S. perpusilla Hook.f. & Thomson	LN812500		LN812737	**LT971182**	Bhutan, Gasa; G. & S. Miehe 00-364-01 (Germany: Marburg)
S. petraea L.	LN812501		LN812738	**LT971183**	Italy, Verona; M. Röser 10236 (HAL)
S. pilifera Hook.f. & Thomson	LN812502		LN812739	**LT971184**	China, Xizang; G. Miehe & U. Wuendisch 94-105-28 (Germany: Marburg)
S. platysepala (Trautv.) Tolm.	LN812503		LN812740	**LT971185**	Russia, Wrangel Island; V.V. Petrovsky (LE)
S. pontica Albov	LN812504		NA	NA	
S. porophylla Bertol.	LN812505		LN812741	**LT971186**	Germany, cult. Brockengarten Harz (99-B-2078); N. Tkach 239 & M. Röser (HAL)
S. portosanctana Boiss.	AJ233883		NA	NA	
S. praetermissa D.A.Webb (1)	LN812506		LN812742	**LT971187**	Spain, Prov. Huesca; M. Röser 10584 (HAL)
S. praetermissa D.A.Webb (2)	**LT970997**	**A**	**LT970896**	**LT971188**	France, Pyrenees-Orient; M. Röser 10612 (HAL)
S. presolanensis Engl.	LN812507		LN812743	NA	
S. przewalskii Engl.	LN812508		LN812744	NA	
S. pseudohirculus Engl.	LN812509		LN812745	**LT971189**	China, Xizang; G. & S. Miehe & U. Wuendisch 94-332-05 (Germany: Marburg)
S. pseudolaevis Oett.	LN812510		NA	NA	
S. pubescens Pourr. subsp. *iratiana* (F.W.Schultz) Engl. & Irmsch.	LN812511		LN812746	**LT971190**	Germany, cult. Brockengarten Harz (04-B-213); N. Tkach 240 & M. Röser (HAL)
S. pulvinaria Harry Sm.	LN812512		LN812747	NA	
S. punctulata Engl.	LM654420		LM654504	**LT971191**	
S. radiata Small (1)	LN812513		LN812748	**LT971192**	USA, Alaska; E. Hulten (LE)
S. radiata Small (2)	LN812418		LN812653	**LT971193**	Russia, Yakutia; V.V. Petrovsky, T.M. Koroleva & M.P. Andreev (LE)
S. retusa Gouan subsp. *augustana* (Vacc.) F.Fournier	LN812514		LN812749	**LT971194**	Germany, cult. Brockengarten Harz (11-B-0145); N. Tkach 241 & M. Röser (HAL)
S. reuteriana Boiss.	AJ233877		NA	NA	
S. rigoi Freyn ex Porta.	AJ233878		NA	NA	
S. rivularis L. subsp. *rivularis*	LN812517		LN812752	**LT971195**	Russia, Chukotka; V.A. Gavrilyuk & P.K. Gagarin (LE)
S. rivularis L. subsp. *arctolitoralis* (Jurtzev & V.V.Petrovsky) H.Jørg. & Elven	LN812515		LN812750	**LT971196**	Russia, Yakutia; T.M. Zaslavskaya, I.M. Litvinov & T.V. Plieva (LE)
S. rosacea Moench	LN812518		LN812753	**LT971197**	Germany, Bavaria; M. Röser 1115 (HAL)
S. rotundifolia L.	LN812519		LN812754	**LT971198**	Germany, cult. Botanical Garden MLU Halle-Wittenberg; N. Tkach 68

					(HAL)
S. roylei Harry Sm.	LN812520		LN812755	**LT971199**	Germany, cult. Brockengarten Harz (02-B-0041); N. Tkach 227 & M. Röser (HAL)
S. rufescens Balf.f.	LN812521		LN812756	NA	
S. ruprechtiana Manden.	NA		LN812757	**LT971200**	Russia, Dagestan; N.N. Tzvelev & et al. (LE)
S. saginoides Hook.f. & Thomson	LN812522		LN812758	**LT971201**	Bhutan, Gasa; G. & S. Miehe 00-361-11 (Germany: Marburg)
S. sancta Griseb.	LN812523		LN812759	**LT971202**	Greece, Nomos Kavalas; M. Röser 2700 (HAL)
S. sanguinea Franch.	EU158849		NA	NA	
S. scardica Griseb.	LN812524		LN812760	**LT971203**	Germany, cult. Brockengarten Harz (11-B-0144); N. Tkach 242 & M. Röser (HAL)
S. scleropoda Sommier & Levier	LN812525		NA	NA	
S. sediformis Engl. & Irmsch.	LN812526		NA	**LT971204**	China, Yunnan; J. F. Rock 9833 (LE)
S. sedoides L. subsp. *hohenwartii* (Vest & Sternb.) P.Schwarz	LN812527		LN812761	**LT971205**	Italy, Lombardia; M. Röser 10442 (HAL)
S. seguieri Spreng. (1)	LN812528		LN812762	**LT971206**	Switzerland, Wallis; M. Röser 1469 (HAL)
S. seguieri Spreng. (2)	LN812350		LN812584	**LT971207**	Italy, Lombardia; M. Röser 6935 (HAL)
S. seguieri Spreng. (3)	NA	**A, B**	NA	NA	Austria, North Tyrol; L. Schratt (WU0002766)
S. sempervivum C.Koch	LN812529		LN812763	**LT971208**	Germany, cult. Botanical Garden Tuebingen; N. Tkach 255 & M. Röser (HAL)
S. serpyllifolia Pursh subsp. *glutinosa* (Sipliv.) Kozhevn.	LN812530		LN812764	**LT971209**	Russia, Taymyr; Yu.P. Kozhevnikov (LE)
S. serpyllifolia Pursh subsp. *serpyllifolia* Pursh	LN812531		LN812765	**LT971210**	Russia, Chukotka; B.A. Jurtzev (LE)
S. setigera Pursh	LM654421		LM654505	**LT971211**	
S. sibirica L.	LN812532		LN812766	**LT971212**	Russia, Altai; M. Mikhailova (LE)
S. sibthorpii Boiss.	LN812533		LN812767	NA	
S. sieversiana Sternb.	LM654422		LM654506	NA	
S. signatella C.Marquand	LN812534		LN812768	**LT971213**	China, Xizang; G. Miehe & U. Wuendisch 94-167-11 (Germany: Marburg)
S. sinomontana J.T.Pan & Gornall (1)	LN812480		LN812715	**LT971214**	Bhutan, Gasa; G. & S. Miehe 00-329-02 (Germany: Marburg)
S. sinomontana J.T.Pan & Gornall (2)	LN812535		LN812769	**LT971215**	China, Xizang; G. & S. Miehe & U. Wuendisch 94-300-35 (Germany: Marburg)
S. spathularis Brot. (1)	AF087626, AF087596		NA	NA	
S. spathularis Brot. (2)	LN812536		LN812770	**LT971216**	Portugal, Beira Alta; M. Röser 9430 (HAL)
S. spinulosa Adams	LN812537		LN812771	NA	
S. spruneri Boiss.	LN812538		LN812772	**LT971217**	Germany, cult. Brockengarten Harz (94-B-917A); N. Tkach 243 & M. Röser (HAL)
S. squarrosa Sieber	AF087617,		NA	NA	

	AF08758				
S. stella-aurea Hook.f. & Thomson var. *polyadena* Harry Sm.	LN812539		LN812773	**LT971218**	China, Qinghai; G. & S. Miehe 9436/05 (Germany: Marburg)
S. stelleriana Merk ex Ser.	KF196359		KF196388	NA	
S. stolitzkae Duthie ex Engl. & Irmsch.	LN812540		LN812774	**LT971219**	Germany, cult. Brockengarten Harz (92-B-214A); N. Tkach 244 & M. Röser (HAL)
S. stolonifera Meerb.	LN812541		LN812775	**LT971220**	Germany, cult. Botanical Garden Tuebingen; N. Tkach 256 & M. Röser (HAL)
S. stribrnyi Podp.	LN812542		LN812776	**LT971221**	Germany, cult. Arctic-Alpine Garden Chemnitz (AC-1388); N. Tkach 218 & M. Röser (HAL)
S. strigosa Wall. et Ser. var. *ramosa* (Engl. & Irmsch.) H.Chuang	JN102240		JN102289	NA	
S. styriaca Köckinger (1)	**LT971388**	**A, B**	**LT970867**	**LT969525**	Austria, Eastern Alps, Styria, Lower Tauern; N.N. Sst_2.1 (KRAM)
S. styriaca Köckinger (2)	**LT971389**	**A, B**	**LT970868**	**LT969526**	Austria, Eastern Alps, Styria, Lower Tauern; N.N. Sst_2.3 (KRAM)
S. styriaca Köckinger (3)	**LT970998**	**A, B, C**	**LT970897**	**LT969527**	Austria, Eastern Alps, Styria, Lower Tauern; S. Ertl Sst_G31.1 (KRAM0636267)
S. styriaca Köckinger (4)	**LT970999**	**A, B, C**	**LT970898**	**LT969528**	Austria, Eastern Alps, Styria, Lower Tauern; S. Ertl Sst_G31.4 (KRAM0636267)
S. substrigosa J.T.Pan	NA		LN812778	**LT971222**	China, Xizang; G. Miehe & U. Wuendisch 94-177-23 (Germany: Marburg)
S. subverticillata Boiss.	LN812543		LN812777	**LT971223**	Germany, cult. Botanical Garden Muenchen-Nymphenburg (GE-0-M-2009/2412); N. Tkach 262 & M. Röser (HAL)
S. tangutica Engl.	LN812544		LN812779	**LT971224**	China, Xizang; G. Miehe & U. Wuendisch 94-099-20 (Germany: Marburg)
S. tatsienluensis Engl.	EU158845		NA	NA	
S. taygetea Boiss. & Heldr.	LN812545		LN812780	NA	
S. taylorii Calder & Savile	LN812546		LN812781	**LT971225**	Canada, British Columbia; J.A. Calder, D.B.O. Savile & R.L. Taylor 23085 (NY)
S. tenella Wulfen (1)	LN812548		LN812783	**LT971226**	Germany, cult. Botanical Garden MLU Halle-Wittenberg; N. Tkach 69 (HAL)
S. tenella Wulfen (2)	LN812547		LN812782	**LT971227**	Germany, cult. Botanical Garden Tuebingen; N. Tkach 257 & M. Röser (HAL)
S. terektensis Bunge	LN812549		LN812784	**LT971228**	Russia, Sayan Mountains; A. Kuminova, N. Peshkova & S. Linde (LE)
S. thessalica Schott, Nym. & Kotschy	LN812550		LN812785	**LT971229**	Germany, cult. Brockengarten Harz (95-B-1154A); N. Tkach 245 & M. Röser (HAL)
S. tibetica Losinsk.	LN812551		LN812786	**LT971230**	China, Qinghai; G. & S. Miehe 9378/06 (Germany: Marburg)
S. tombeanensis Boiss. ex Engl.	LN812552		LN812787	NA	
S. trabutiana Engl. & Irmsch.	LN812553		NA	NA	
S. tricuspidata Rottb. (1)	LN812554		LN812788	**LT971231**	Germany, cult. Rennsteiggarten (3/85/338); N. Tkach 205 & M. Röser (HAL)

S. tricuspidata Rottb. (2)	LN812555		LN812789	**LT971232**	USA, Alaska; M.H. Hoffmann 11/22 (HAL)
S. tridactylites L. (1)	LN812556	**A**	LN812790	**LT971233**	Germany, Baden-Wuerthemberg; M. Röser 9927 (HAL)
S. tridactylites L. (2)	**LT971000**	**A, B**	**LT970899**	**LT971234**	Turkmenistan, Kopet-Dag; V.V. Nikitin & M. Batyrova (LE)
S. trifurcata Schrad.	LN812557		LN812791	**LT971235**	Germany, cult. Arctic-Alpine Garden Chemnitz (AC-1309); N. Tkach 220 & M. Röser (HAL)
S. umbellulata Hook.f. & Thomson var. *umbellulata*	LN812559		LN812793	**LT971236**	China, Xizang; G. Miehe & U. Wuendisch 94-156-26 (Germany: Marburg)
S. umbellulata Hook.f. & Thomson var. *muricola* (C.Marquand & Airy Shaw) J.T.Pan	LN812558		LN812792	**LT971237**	China, Xizang; G. Miehe & U. Wuendisch 94-055-20 (Germany: Marburg)
S. umbellulata Hook.f. & Thomson var. *pectinata* (C.Marquand & Airy Shaw) J.T.Pan	EU158832		NA	NA	
S. umbrosa L.	LN812560		LN812794	**LT971238**	Germany, cult. Botanical Garden MLU Halle-Wittenberg; N. Tkach 72 (HAL)
S. unguiculata Engl.	LN812561		LN812795	**LT971239**	China, Xizang; G. & S. Miehe 9497/17 (Germany: Marburg)
S. unguipetala Engl. & Irmsch.	LN812562		LN812796	NA	
S. valdensis DC. (1)	LN812563		LN812797	NA	
S. valdensis DC. (2)	AF087612, AF087582		NA	NA	
S. vandellii Sternb.	LN812564		LN812798	**LT971240**	Germany, cult. Brockengarten Harz (07-B-0031); N. Tkach 247 & M. Röser (HAL)
S. vayredana Luizet	LN812565		LN812799	**LT971241**	Germany, cult. Botanical Garden Muenchen-Nymphenburg (XX-0-MG/0019); N. Tkach 248 & M. Röser (HAL)
S. veitchiana Balf.f.	LN812566		LN812800	**LT971242**	Germany, cult. Arctic-Alpine Garden Chemnitz (AC-1366); N. Tkach 221 & M. Röser (HAL)
S. vespertina (Small) Fedde	KF196350		KF196392	NA	
S. viscidula Hook.f. & Thomson	LN812567		LN812801	NA	
S. wahlenbergii Ball (1)	LN812568		LN812802	NA	
S. wahlenbergii Ball (2)	**LT971003**	**A, B, C**	**LT970902**	**LT969537**	Poland, Carpathians, Western Tatras; R.Letz, P. Mraz (KRAM0636268) SWA_747.2
S. wahlenbergii Ball (3)	**LT971002**	**A, B**	**LT970901**	LT969536	Poland, Carpathians, High Tatras; M. Ronikier (KRAM0636266) SWA_648.1
S. wahlenbergii Ball (4)	NA	**A, B**	NA	**LT969536**	Poland, Carpathians, High Tatras; M. Ronikier (KRAM) SWA_648.2
S. wahlenbergii Ball (5)	**LT971006**	**A, B, C**	**LT970904**	**LT969529**	Poland, Carpathians, High Tatras; A. Delimat (KRAM) SWA_K1
S. wahlenbergii Ball (6)	**LT971008**	**A**	**LT970906**	**LT969531**	Poland, Carpathians, Western Tatras; A. Delimat (KRAM) SWA_P1
S. wahlenbergii Ball (7)	**LT971009**		**LT970907**	**LT969532**	Slovakia, Carpathians, Little Fatra; M. Ronikier (KRAM) SWA_R1
S. wahlenbergii Ball (8)	**LT971010**	**A**	**LT970908**	**LT969533**	Slovakia, Carpathians, Little Fatra; M. Ronikier (KRAM) SWA_R5
S. wahlenbergii Ball (9)	**LT971004**	**A, B**	**LT970903**	**LT969538**	Slovakia, Carpathians, Lower Tatras; R. Letz, P. Turis

					(KRAM0636261) SWA_846.2
S. wahlenbergii Ball (10)	**LT971007**	**A, B**	**LT970905**	**LT969530**	Poland, Carpathians, High Tatras; A. Delimat (KRAM) SWA_M1
S. wahlenbergii Ball (11)	**LT971001**	**A**	**LT970900**	**LT969535**	Slovakia, Carpathians, Choč Mountains; M. Ronikier (KRAM) SWA_V10
S. wahlenbergii Ball (12)	**LT971005**		NA	**LT971243**	Slovakia, Carpathians, Belianske Tatras; M. Deyl (HAL0010087)
S. wahlenbergii Ball (13)	**LT971011**	**A, B**	**LT970909**	**LT969534**	Slovakia, Carpathians, Choč Mountains; M. Ronikier (KRAM) SWA_V9
S. wahlenbergii Ball (14)	**LT971390**	**A, B, C**	**LT970866**	**LT969524**	Slovakia, Carpathians, High Tatras; M. Ronikier (KRAM) SWA_H1
S. wallichiana Sternb.	LN812569		LN812803	**LT971244**	Bhutan, Thimphu; G. & S. Miehe 00-228-31 (Germany: Marburg)
S. wardii W.W.Sm.	LN812570		LN812804	**LT971245**	China, Xizang; G. Miehe & U. Wuendisch 94-173-07 (Germany: Marburg)
S. wendelboi Schönb.-Tem.	LN812571		LN812805	**LT971246**	Germany, cult. Arctic-Alpine Garden Chemnitz (AC-1524); N. Tkach 222 & M. Röser (HAL)
S. xiaozhongdianensis J.T.Pan	EU158835		NA	NA	
S. yarlungzangboensis J.T.Pan	LN812572		LN812806	**LT971247**	China, Xizang; G. Miehe & U. Wuendisch 94-166-06 (Germany: Marburg)
S. zhidoensis J.T.Pan	EU158862		NA	NA	
S. zimmermannii Baehni	LN812573		LN812807	**LT971248**	Nepal, Langtang Valley; U. Wuendisch 278 (Germany: Marburg)
Saxifragodes albowiana (Kurtz ex Albov) D.M.Moore	AF374825, AF374826		NA	NA	
Saxifragopsis fragarioides (Greene) Small	JQ895204		AF374815	NA	
Suksdorfia violacea A.Gray	U51257		NA	NA	
Sullivantia oregana S.Watson	U51258		AF374811	NA	
Tanakaea radicans Franchet & Savatier	U51263		AF374813	NA	
Telesonix heucheriformis (Rydb.) Rydb.	U51260		AF374807	NA	
Tellima grandiflora (Pursh) Douglas ex Lindl.	AF158952		AF374805	KT755743	
Tiarella polyphylla D.Don	AB163608, AB163671		AB116717	NA	
Tolmiea menziesii (Pursh) Torr. & A.Gray	AB248857		AF374803	KT755744	

[1] Xiang C-L, Gitzendanner MA, Soltis DE, Peng H, Lei L.G. Phylogenetic placement of the enigmatic and critically endangered genus *Saniculiphyllum* (Saxifragaceae) inferred from combined analysis of plastid and nuclear DNA sequences. *Molec Phylogen Evol.* 2012;64: 357-367. doi:10.1016/j.ympev.2012.04.010
