# Supplementary material for: Contrasting evolutionary origins of two mountain endemics: Saxifraga wahlenbergii (Western Carpathians) and S. styriaca (Eastern Alps)
Source: BMC Evol Biol. 2019 Jan 11;19:18. doi: 10.1186/s12862-019-1355-x (PMC6329101; doi:10.1186/s12862-019-1355-x)
Supplement: Supplementary file 3 — Figure S1. Maximum likelihood phylogram of Saxifraga (arrow) and representative genera of Saxifragaceae based on nuclear ribosomal ITS DNA Sanger sequence data. Pterostemon rotundifolius and Itea virginica (Iteaceae) were chosen as outgroups. Maximum likelihood and maximum parsimony bootstrap support values as well as posterior probabilities of Bayesian inference ≥50% are indicated on the branches. The sections of Saxifraga are labelled on the right-hand side. (PDF 2680 kb) [file 12862_2019_1355_MOESM3_ESM.pdf]

Additional file 3

Contrasting evolutionary origins of two mountain endemics: *Saxifraga wahlenbergii* (Western Carpathians) and *S. styriaca* (Eastern Alps)

Natalia Tkach[1*¶], Martin Röser[1¶], Tomasz Suchan[2], Elżbieta Cieślak[2], Peter Schönswetter[3], Michał Ronikier[2*]

[1] Martin Luther University Halle-Wittenberg, Institute of Biology, Geobotany and Botanical Garden, Neuwerk 21, 06108 Halle, Germany

[2] W. Szafer Institute of Botany, Polish Academy of Sciences, Lubicz 46, 31-512, Krakow, Poland

[3] University of Innsbruck, Department of Botany, Sternwartestraße 15, 6020 Innsbruck, Austria

* Corresponding authors: m.ronikier@botany.pl, natalia.tkach@botanik.uni-halle.de

[¶] These authors contributed equally to this work.

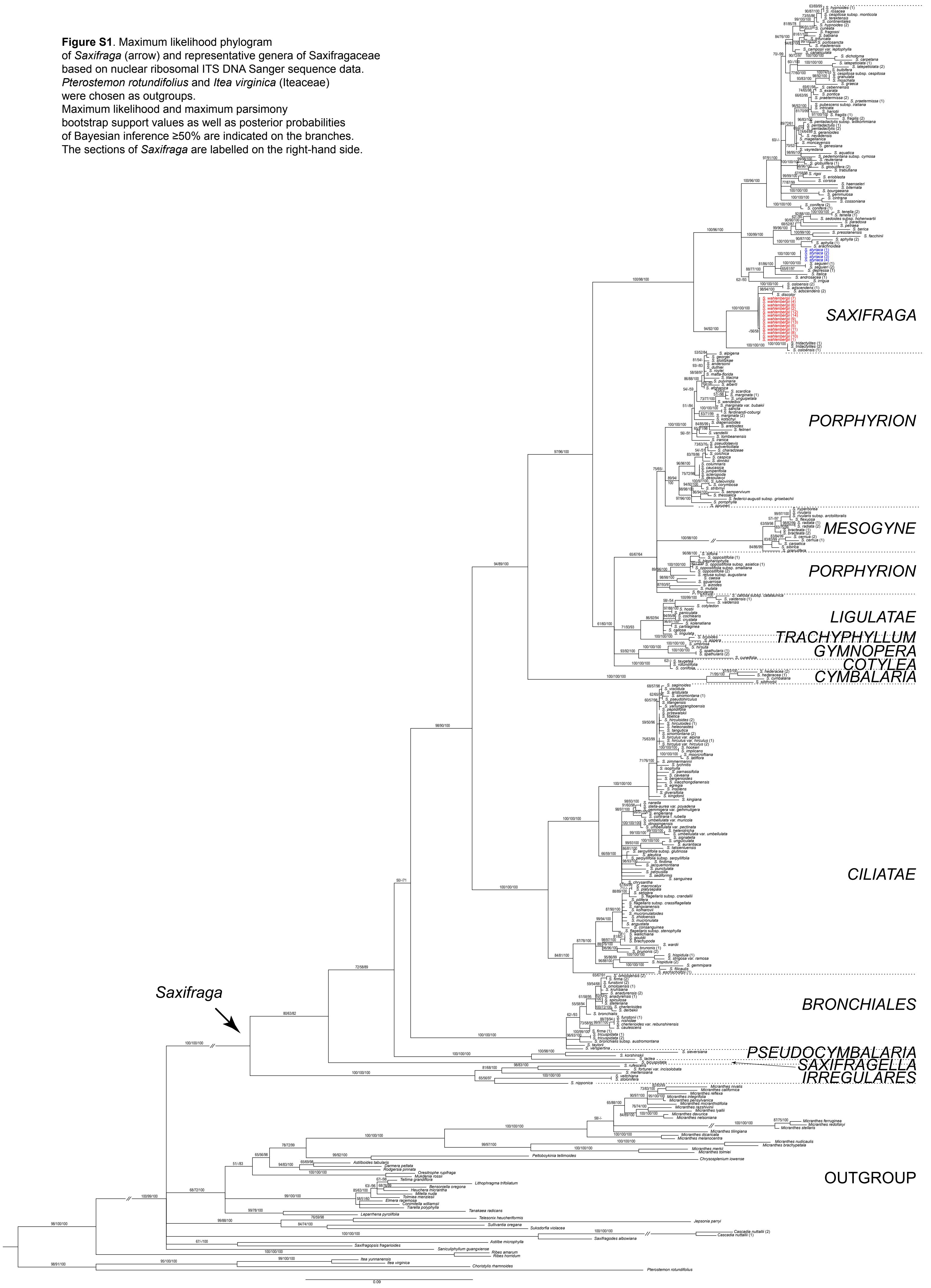

Figure S1. Maximum likelihood phylogram of *Saxifraga* (arrow) and representative genera of Saxifragaceae based on nuclear ribosomal ITS DNA Sanger sequence data. *Pterostemon rotundifolius* and *Itea virginica* (Iteaceae) were chosen as outgroups. Maximum likelihood and maximum parsimony bootstrap support values as well as posterior probabilities of Bayesian inference ≥50% are indicated on the branches. The sections of *Saxifraga* are labelled on the right-hand side.
