# Supplementary material for: Contrasting evolutionary origins of two mountain endemics: Saxifraga wahlenbergii (Western Carpathians) and S. styriaca (Eastern Alps)
Source: BMC Evol Biol. 2019 Jan 11;19:18. doi: 10.1186/s12862-019-1355-x (PMC6329101; doi:10.1186/s12862-019-1355-x)

Additional file 4

Contrasting evolutionary origins of two mountain endemics: *Saxifraga wahlenbergii* (Western Carpathians) and *S. styriaca* (Eastern Alps)

Natalia Tkach[1*¶], Martin Röser[1¶], Tomasz Suchan[2], Elżbieta Cieślak[2], Peter Schönswetter[3], Michał Ronikier[2*]

[1] Martin Luther University Halle-Wittenberg, Institute of Biology, Geobotany and Botanical Garden, Neuwerk 21, 06108 Halle, Germany

[2] W. Szafer Institute of Botany, Polish Academy of Sciences, Lubicz 46, 31-512, Krakow, Poland

[3] University of Innsbruck, Department of Botany, Sternwartestraße 15, 6020 Innsbruck, Austria

* Corresponding authors: m.ronikier@botany.pl, natalia.tkach@botanik.uni-halle.de

¶ These authors contributed equally to this work.

Figure S2. Maximum likelihood phylogram of *Saxifraga* (arrow) and representative genera of Saxifragaceae based on plastid *trn*L–*trn*F and *rpl*32–*trn*L Sanger sequence data. *Pterostemon rotundifolius* and *Itea virginica* (Iteaceae) were chosen as outgroups. Maximum likelihood and maximum parsimony bootstrap support values as well posterior probabilities of Bayesian inference ≥50% are indicated on the branches. The sections of *Saxifraga* are labelled on the right-hand side.

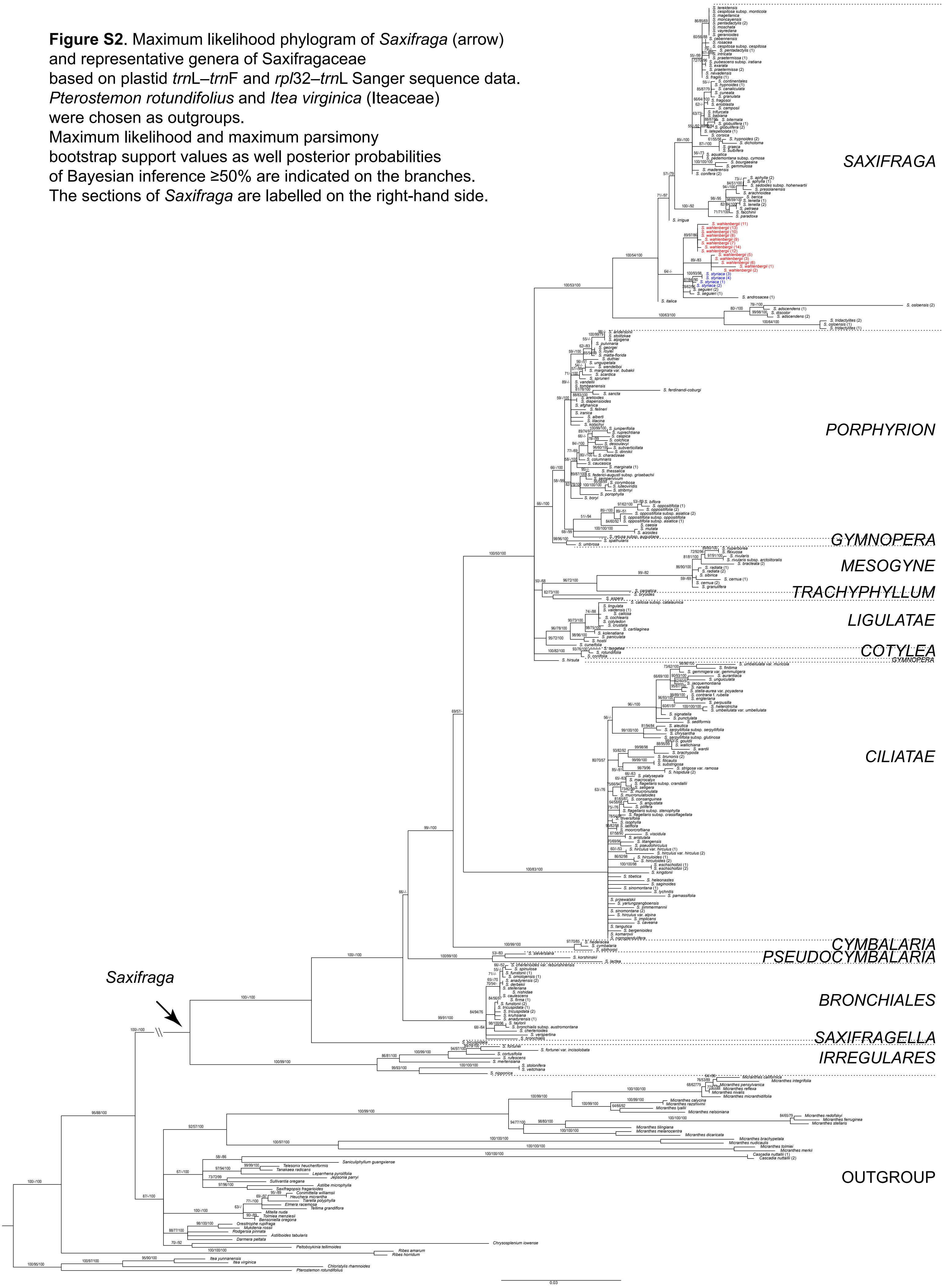

Supplement: Supplementary file 4 — Figure S2. Maximum likelihood phylogram of Saxifraga (arrow) and representative genera of Saxifragaceae based on plastid trnL–trnF and rpl32–trnL Sanger sequence data. Pterostemon rotundifolius and Itea virginica (Iteaceae) were chosen as outgroups. Maximum likelihood and maximum parsimony bootstrap support values as well posterior probabilities of Bayesian inference ≥50% are indicated on the branches. The sections of Saxifraga are labelled on the right-hand side. (PDF 793 kb) [file 12862_2019_1355_MOESM4_ESM.pdf]
